# Supplementary material for: Deep recurrent reinforced learning model to compare the efficacy of targeted local versus national measures on the spread of COVID-19 in the UK
Source: BMJ Open. 2022 Feb 21;12(2):e048279. doi: 10.1136/bmjopen-2020-048279 (PMC8861888; doi:10.1136/bmjopen-2020-048279)
Supplement: Author's manuscript [file bmjopen-2020-048279.draft_revisions.pdf]

BMJ Open is committed to open peer review. As part of this commitment we make the peer review history of every article we publish publicly available.

When an article is published we post the peer reviewers' comments and the authors' responses online. We also post the versions of the paper that were used during peer review. These are the versions that the peer review comments apply to.

The versions of the paper that follow are the versions that were submitted during the peer review process. They are not the versions of record or the final published versions. They should not be cited or distributed as the published version of this manuscript.

BMJ Open is an open access journal and the full, final, typeset and author-corrected version of record of the manuscript is available on our site with no access controls, subscription charges or pay-per-view fees (http://bmjopen.bmj.com).

If you have any questions on BMJ Open's open peer review process please email info.bmjopen@bmj.com

BMJ Open

# A Deep Recurrent Reinforced Learning model to compare the efficacy of targeted local vs. national measures on the spread of COVID-19 in the UK



| | |
|---|---|
| Journal: | *BMJ Open* |
| Manuscript ID | bmjopen-2020-048279 |
| Article Type: | Original research |
| Date Submitted by the Author: | 26-Dec-2020 |



| | |
|---|---|
| Complete List of Authors: | Dong, Tim; University of Bristol, Bristol Heart Institute, Bristol Medical School<br>Benedetto, Umberto; University of Bristol, Bristol Heart Institute, Bristol Medical School<br>Sinha, Shubhra ; University of Bristol, Bristol Heart Institute, Bristol Medical School<br>Dimagli, Arnaldo ; University of Bristol, Bristol Heart Institute, Bristol Medical School<br>Caputo, Massimo; University of Bristol, Bristol Heart Institute, Bristol Medical School<br>Angelini, Gianni; University of Bristol, Bristol Heart Institute, Bristol Medical School |



| | |
|---|---|
| Keywords: | COVID-19, VIROLOGY, Infection control < INFECTIOUS DISEASES, Health informatics < BIOTECHNOLOGY & BIOINFORMATICS, Health policy < HEALTH SERVICES ADMINISTRATION & MANAGEMENT |


SCHOLARONE™
Manuscripts

*I, the Submitting Author has the right to grant and does grant on behalf of all authors of the Work (as defined in the below author licence), an exclusive licence and/or a non-exclusive licence for contributions from authors who are: i) UK Crown employees; ii) where BMJ has agreed a CC-BY licence shall apply, and/or iii) in accordance with the terms applicable for US Federal Government officers or employees acting as part of their official duties; on a worldwide, perpetual, irrevocable, royalty-free basis to BMJ Publishing Group Ltd ("BMJ") its licensees and where the relevant Journal is co-owned by BMJ to the co-owners of the Journal, to publish the Work in this journal and any other BMJ products and to exploit all rights, as set out in our licence.*

*The Submitting Author accepts and understands that any supply made under these terms is made by BMJ to the Submitting Author unless you are acting as an employee on behalf of your employer or a postgraduate student of an affiliated institution which is paying any applicable article publishing charge ("APC") for Open Access articles. Where the Submitting Author wishes to make the Work available on an Open Access basis (and intends to pay the relevant APC), the terms of reuse of such Open Access shall be governed by a Creative Commons licence – details of these licences and which Creative Commons licence will apply to this Work are set out in our licence referred to above.*

*Other than as permitted in any relevant BMJ Author's Self Archiving Policies, I confirm this Work has not been accepted for publication elsewhere, is not being considered for publication elsewhere and does not duplicate material already published. I confirm all authors consent to publication of this Work and authorise the granting of this licence.*

For peer review only

# A Deep Recurrent Reinforced Learning model to compare the efficacy of targeted local vs. national measures on the spread of COVID-19 in the UK

*Tim Dong[1†] (0000-0003-1953-0063), Umberto Benedetto (0000-0002-7074-7949)[2†*], Shubhra Sinha[3], Arnaldo Dimagli[4], Massimo Caputo[5], Gianni D Angelini[6],*

[1] Assistant Database Manager, Bristol Heart Institute, Bristol Medical School, University of Bristol BS2 8DZ, United Kingdom

[2] Associate Professor in Cardiac Surgery, Bristol Heart Institute, Bristol Medical School, University of Bristol BS2 8DZ, United Kingdom

[3] National Trainee Number in Cardiothoracic Surgery, Bristol Heart Institute, Bristol Medical School, University of Bristol BS2 8DZ, United Kingdom

[4] Assistant Database Manager, Bristol Heart Institute, Bristol Medical School, University of Bristol BS2 8DZ, United Kingdom

[5] Professor of Congenital Heart Surgery, Bristol Heart Institute, Bristol Medical School, University of Bristol BS2 8DZ, United Kingdom

[6] BHF Professor of Cardiac Surgery, Bristol Heart Institute, Bristol Medical School, University of Bristol BS2 8DZ, United Kingdom

[*] Corresponding Author; umberto.benedetto@bristol.ac.uk; +44 (0) 117 3428854

[†] TD and UB contributed equally to this paper



For peer review only - http://bmjopen.bmj.com/site/about/guidelines.xhtml

# ABSTRACT

**Objectives** We have developed a deep learning model that provides predictions of the COVID-19 related number of cases and mortality in the upcoming 5 weeks and simulates the effect of policy changes targeting COVID-19 spread.

**Methods** We developed a Deep Recurrent Reinforced Learning (DRRL) based model. The data used to train the DRRL model was based on various available datasets that have the potential to influence the trend in the number of COVID-19 cases and mortality. Analyses were performed based on the simulation of policy changes targeting COVID-19 spread, and the geographical representation of these effects.

**Results** Model predictions of the number of cases and mortality of COVID-19 in the upcoming 5 weeks closely matched the actual values. Local lockdown with social distancing (LD_SD) was found to be ineffective compared to national lockdown. The ranking of effectiveness of supplementary measures for LD_SD were found to be consistent across national hotspots and local areas. Measure effectiveness were ranked from most effective to least effective: 1) full lockdown; 2) LD_SD with international travel -50%; 3) LD_SD with 100% quarantine; 4) LD_SD with closing school -50%; 5) LD_SD with closing pubs -50%. There were negligible differences observed between LD_SD, LD_SD with -50% food & Accommodation and LD_SD with -50% Retail.

**Conclusions** The second national lockdown should be followed by measures which are more effective than LD_SD alone. Our model suggests the importance of restrictions on international travel and travel quarantines, thus suggesting that follow-up policies should consist of the combination of LD_SD and a reduction in the




For peer review only - http://bmjopen.bmj.com/site/about/guidelines.xhtml

number of open airports within close proximity of the hotspot regions. Stricter measures should be placed in terms travel quarantine to increase the impact of this measure. It is also recommended that restrictions should be placed on the number of schools and pubs open.

**Strengths and limitations of this study**

- The proposed Deep Recurrent Reinforced Learning (DRRL)-based model takes into account of both relationships of variables across local authorities and across time, using ideas from reinforcement learning to improve predictions.
- Whilst, predicting the geographical trend in COVID-19 cases based on the simulation of different measures in the UK at both the national and local levels in the UK has proved challenging, this study has provided a methodology by which useful predictions and simulations can be obtained.
- The Office for National Statistics only released data on UK international travel up to March 2019 at the time of this study, and therefore this study used the amount of UK tourists in Spain as a reference variable for understanding the effect of international travel on COVID-19 spread.



For peer review only - http://bmjopen.bmj.com/site/about/guidelines.xhtml

# INTRODUCTION

COVID-19 is a highly infectious disease that resulted in a global pandemic in just under a month. This pandemic has caused global disruptions to individuals, businesses and governments worldwide. The number of cases have continued to rise exponentially, from 80,239 in February 2020 rising to 69 million as of December 2020.[1] Recent cases of a new variants of COVID-19 have also been found [2].This is despite global efforts to control this virus. Thus, novel strategies are necessary to monitor and control the spread of this virus.

Whilst there have been studies of the geographical distribution of actual COVID-19 cases[3], to the best of our knowledge, there have not been any studies predicting the geographical trend in COVID-19 cases based on the simulation of different interventions at both the national and local authority(LA) levels in the UK. It is vital to have a detailed understanding of the factors that presently affect the spread of COVID-19 at both a national and local level as well as the potential impact of future policy measures. This knowledge would allow the government, LAs and individual citizens to make informed decisions about regional policies and personal exposure risks.

To this end, we have developed a deep learning model that provides the predictions of the incidence and mortality related to COVID-19 in the upcoming 5 weeks and simulates the effect of policy changes targeting the COVID-19 spread i.e. number of facilities available for accommodation and food, pubs, retail shops, education, transport and storage, art, entertainment and recreational services, within each local authority region. The model also accounts for international migration



For peer review only - http://bmjopen.bmj.com/site/about/guidelines.xhtml

inflow, internal migration inflow and outflow within the UK thus simulating policy changes that affect travel. This model may also inform planning for similar scenarios in the future.

## METHODS

### Patient and public involvement

This research was done without patient and public involvement.

### Model Development

We developed a Deep Recurrent Reinforced Learning (DRRL)-based model (supplementary material Part I) that combines the synergistic properties of Gated Recurrent Units (GRU),[4] and reinforcement deep learning.[5] We have chosen GRU as an element of our model because of its ability to model non-linear and temporal relationships between and within high dimensions of variables. The reinforcement learning element of DRRL enables it to adapt to newly inputted data and make more accurate forecasts.

All available LA data was split 80:20 into training and validation data subsets. Data was pre-processed using scaling - subtracting their corresponding mean and dividing by the standard deviation values.  Following the completion of predictions, the prediction outputs are then scaled back to their original scale.

The DRRL neural network model utilised an input layer, numerous hidden layers, and an output layer. A complex series of non-linear matrix computations are applied to the input data to relate the target output (i.e. cases and mortality) to the



For peer review only - http://bmjopen.bmj.com/site/about/guidelines.xhtml

other data columns (e.g. amount of international migration inflow or internal migration inflow and outflow within UK, number of retail shops etc.). The model is first Trained using the existing data subsets. Each column of the data is assigned a specific weight at each of the nodes in the hidden layers and these weights are progressively updated to minimize the the mean absolute error (MAE) between the predicted and actual values, using the rmsprop optimisation algorithm.[6] During the Prediction process, an input data matrix of the same dimension as the training data is then passed into the input layer. The neural network's hidden layers then use the weights learned during training process to predict the most likely incidence and mortality based on the values of the other variables from each corresponding week.

The final model consists of two components, model-M (master model) and model-R (reinforced model) that serve different purposes. Model-M accounts for the relationships of variables across different LAs, whilst model-R provides improved forecasting performance for each individual LA that are selected for analysis (see Supplementary Material, Part I). This model is particularly apt at generalisation and is capable of forecasting a wide range of LA simultaneously. The model uses model-R to increase forecast performance for the individual LA that are selected for analysis. Model-M is updated with several additional epochs of training data from the selected LA to reinforce and optimise the predictions.

## Data Linkage

The data used to train the deep learning model is based on various datasets that have the potential to influence the trend in the number of COVID-19 cases and mortality at the national and local level (refer to Supplementary Materials, Part II. for more details), including domains of deprivation, number of bars and pubs, business



For peer review only - http://bmjopen.bmj.com/site/about/guidelines.xhtml

size, population estimate (male, female, by age, overall), etc. We use the R language and the R SDK for COVID-19, i.e. a set of software commands to retrieve data remotely, as published by Public Health England, to automatically extract the latest daily cases and mortality figures for all LA within the UK. Using this approach, we are able to automate and dynamically predict the cases and mortality as new data is generated by GOV.UK. We use R to convert these data from daily figures into weekly counts and link this data to the data described in Supplementary Materials, Part II.

Specifically, we have created a manually curated datasheet containing three indices that together we name the COVID-19 General Policy (CvdGPlc) indices: LockdownScore, QuarantineMeasures and SchoolOpening. These index scores are linked to the main dataset based on the weeks each of the corresponding policies were implemented and the relative effects at each time period.

Furthermore, we obtained the number of tourists arriving in Spain from Jan 2020 to July 2020 and adjusted the number by the proportion of UK tourists in Spain from the year 2019. As data on international travel is not readily available for the period affected by COVID-19, the rationale is to use the amount of UK travel to Spain as an indicator for the impact of international travel on the spread of COVID-19, since Spain is a frequent UK tourist destination. Our GRU model is not only trained on the above data, but also includes the longitude and latitude of each local authority as part of the model.

In this study, we have explored the effects of various containing measures at specific time periods to investigate their effects on virus spread and mortality rates. Only the variables that we have found most relevant as measures for policy making have been reported. The reader is encouraged to explore the adjustment effects of



For peer review only - http://bmjopen.bmj.com/site/about/guidelines.xhtml

other variables on the prediction results of the model via our online web app (http://137.222.198.54:8081/).

Whilst the LA boundaries data is not included in the training process, the main dataset is also linked to this data following forecast generation, so the deep learning model will also provide the prediction of the incidence or mortality in the next five weeks in a geographical map view. Furthermore, the model has the capability to toggle the map view by local authority or Public Health England regions. These views will not only be useful for the government to see the future effects of different policy changes, but also for the individual citizens to understand their risk of movement within and between local regions in the upcoming future.

For analysis in the map view, the geographical regions from the top to bottom of England is divided into four equidistant slices, which we shall name slice n2, n1, s1, s2, respectively. These categories will be applied to all other geographical plots hereafter to facilitate discussion. The areas with higher number of cases are shown in darker colours with 6 grades of severity (I – VI) covering the ranges 0-250 (I); 250-500 (II); 500-750 (III); 750-1000 (IV); 1000-1250 (V); 1250-1500 (VI). Any number outside of this range is shown in grey and is classed as grade VII.

## Model Validation

The model is validated for the whole of England, whereby the model is trained using the same approach as described in the Model Development section but excluding data from weeks 41 to 46. The data from this interval serves as Validation data for determining the performance of the model on unseen data. Ten iterations of reinforced training are performed over the dataset. At the time of this work, only data up to week 46 are available. The variable values are set from week 40 onwards to



For peer review only - http://bmjopen.bmj.com/site/about/guidelines.xhtml

enable predictions to simulate a full/national lockdown (FLD) from week 45 onwards. This is because it is known that a FLD had been applied in the UK from week 45 (00.01 on Thursday 5 November) and prior to that, local lockdown with social distancing (LD_SD) had been implemented.

## Model Simulation

Simulations are performed using the final model that is trained using the approach described in the Model Development section. All data i.e. from week 1 to 46 are included for training this model. The model is used to simulate the effects of numerous different COVID-19 prevention measures on the number of cases at week 51 i.e. 5 weeks ahead of the latest available data. The values of the variables that model the corresponding measures are set from week 40 onwards to enable predictions to simulate the implementation of those measures from week 45 onwards, rather than FLD, which was what the government actually implemented. The measures simulated are: (a) No lockdown vs. local lockdown with social distancing (LD_SD); b) LD_SD vs. full/national lockdown (FLD); c) LD_SD vs. LD_SD with international travel -50%; d) LD_SD vs. LD_SD with closing school -50% e) LD_SD with travel quarantine 5.5 (see Supplementary Material, Part II., 11) vs. LD_SD with full travel quarantine 10; f) LD_SD with 100% pubs open vs. LD_SD with -50% pubs; (g) LD_SD with 100% food & accommodation services open vs. LD_SD with -50% food & accommodation services open; (h) LD_SD with -50% retail services open vs. LD_SD with 100% retail services open. For details on the implementation of these measures, please refer to Supplementary Materials, Part II.

These measures are simulated firstly for individual LA by selecting a baseline LA with a relatively low case count and comparing the effect of the measures when



For peer review only - http://bmjopen.bmj.com/site/about/guidelines.xhtml

applied to a LA with a very high number of cases i.e. a hotspot area. The measures are then ranked by order of effectiveness. This is so that the relative effectiveness of each measure can be understood at the local level. Secondly, the measures are simulated for all the LA in England to visualise the relative effectiveness of each measure at a national level. For the 21 LA with the highest cases when using a LD_SD measure, the predicted cases counts at week 51 are extracted and plotted to analyse the efficacy of each measure across these nationally “hard” to tackle areas. This comparison also enabled the ranking of the relative effectiveness of each measure at these hotspots.

## RESULTS

Model validation of predictions against actual results for week 46 showed a good match between the simulation and actual number of cases across all the LA concerned (fig 1). The model was able to distinguish LA with high cases from areas with low number of cases (fig 2a, b). The model performs especially well for low grade LA (Table 1). The tendency towards better performance in low degree LA, may be because data from week 41 to 46 containing sharp changes in the trend have not been included. Therefore, the simulation of cases and mortality up to week 51 was performed by including data from week 41 to 46 in the model training.

The effects of different measures were first observed at a local level. Southampton was selected as baseline for observing the effects of measure changes. As Southampton is a grade I LA with a low case number of 187 in week 46, the effects of measure changes were readily perceived with effectiveness ranked from most effective to least effective (Supplementary Materials, Part IV fig. S6): b) full lockdown;



For peer review only - http://bmjopen.bmj.com/site/about/guidelines.xhtml

c) LD_SD & international travel -50%; e) LD_SD & 100% quarantine; d) LD_SD & closing school -50%; f) LD_SD & closing pubs -50%. There were negligible differences observed between LD_SD, g) LD_SD & -50% food & Accommodation and h) LD_SD & -50% Retail.

As Leeds was in the highest grade (VII) for both week 46 (actual) and week 51 (predicted), it was selected for observing the effects of different measures on ‘hard’ to tackle areas. As the number of cases for Leeds were approximately 5 times higher than Southampton, the effect of measures relative to the number of cases in any week were much smaller in the former compared to the latter. For Leeds, no difference was observed for predicted cases at week 51 between no lockdown and LD_SD. Full lockdown (Supplementary Materials, Part IV. fig. S7b) was most effective followed by LD_SD with a reduction in international travel by 50%, although the effects were much less in proportion to the number of cases than Southampton. There was negligible impact on the number of cases at week 51 for the remaining measures (fig. S7e-h).

Figure 2c shows the predicted cases in week 51 using LD_SD. At a national level, it can be seen that there would be a rapid rise in the number of cases, especially in the horizontal “belt” along the n1 region. In addition, there is at least one LA in each of the other slices n2, s1 and s2 that are expected to rise to grade VI or above. The majority of LA locations elsewhere, which were mostly at grade I in week 46, are expected to rise to grade II or III. The top 21 hotspots at week 51 using LD_SD were selected for subsequent analysis (Table 2).

LD_SD was shown (fig 3) to be effective in suppressing the increase in cases for Birmingham (-17%), Bradford (+0.98%), Kirklees (-6.6%) and Leicester (-1.3%). LD_SD was shown to be ineffective for suppressing the increase in cases for the



For peer review only - http://bmjopen.bmj.com/site/about/guidelines.xhtml

remaining 17 LA, with the highest predicted rises for Wirral (325%), Stockport (163%), Tameside (188%), Rotherham (158%), Derby (130%).

**Table 1 Validation model: Number of actual and predicted cases and mortalities. The results show that there is a close match between actual and predicted number of cases, especially for LA at grade III or below.**

| Local Authority | Number of Actual Cases for week 46 | Cases Forecast for week 46 | Number of Actual Mortalities for week 46 | Mortality Forecast for week 46 |
|---|---|---|---|---|
| Intervention | *Full Lockdown* | | | |
| Wolverhampton | 438 | 482 | 4 | 5 |
| Gedling | 179 | 196 | 6 | 2 |
| Welwyn Hatfield | 119 | 130 | 0 | 2 |
| Wiltshire | 201 | 219 | 1 | 4 |
| Portsmouth | 220 | 239 | 0 | 3 |
| Bromley | 217 | 232 | 2 | 3 |
| Stockton-on-Tees | 467 | 498 | 7 | 7 |
| Stockport | 517 | 550 | 13 | 8 |
| South Kesteven | 153 | 162 | 6 | 1 |
| Hammersmith and Fulham | 166 | 175 | 1 | 2 |
| Kingston upon Thames | 150 | 158 | 4 | 2 |
| Ribble Valley | 93 | 98 | 4 | 2 |
| East Cambridgeshire | 34 | 36 | 1 | 1 |
| Redcar and Cleveland | 380 | 396 | 7 | 4 |
| Sedgemoor | 55 | 57 | 3 | 1 |
| Cheshire East | 496 | 514 | 6 | 7 |
| Wealden | 76 | 79 | 1 | 2 |
| Charnwood | 371 | 382 | 3 | 3 |
| South Somerset | 72 | 74 | 1 | 1 |
| Southend-on-Sea | 137 | 140 | 0 | 3 |
| Chelmsford | 110 | 112 | 2 | 2 |
| Rushcliffe | 124 | 126 | 4 | 2 |
| Merton | 146 | 148 | 0 | 2 |
| Shropshire | 426 | 428 | 6 | 6 |
| Harrogate | 253 | 253 | 1 | 2 |
| Central Bedfordshire | 226 | 225 | 5 | 4 |
| Sutton | 155 | 154 | 5 | 3 |
| Oldham | 735 | 732 | 15 | 8 |
| Hillingdon | 325 | 323 | 3 | 3 |
| Basildon | 168 | 167 | 4 | 3 |
| Plymouth | 196 | 192 | 2 | 3 |
| Test Valley | 59 | 58 | 1 | 1 |
| Walsall | 605 | 590 | 15 | 6 |

For peer review only

| | | | | |
|---|---|---|---|---|
| Southampton | 187 | 182 | 0 | 2 |
| Selby | 129 | 124 | 2 | 1 |
| South Holland | 105 | 100 | 2 | 1 |
| Chiltern | 57 | 54 | 0 | 1 |
| Derbyshire Dales | 82 | 78 | 1 | 2 |
| Chichester | 58 | 54 | 0 | 1 |
| Barnet | 378 | 354 | 5 | 4 |
| Tameside | 447 | 417 | 18 | 12 |
| Salford | 577 | 537 | 17 | 7 |
| Havant | 77 | 71 | 1 | 1 |
| Waverley | 97 | 89 | 0 | 1 |
| Nuneaton and Bedworth | 247 | 226 | 4 | 3 |
| New Forest | 92 | 84 | 6 | 1 |
| Ryedale | 67 | 61 | 1 | 1 |
| Peterborough | 224 | 204 | 2 | 4 |
| North Hertfordshire | 89 | 81 | 1 | 1 |
| Epping Forest | 130 | 118 | 2 | 1 |

Note: only 50 LA are displayed. For validation data on all LA, please contact the authors.

For peer review only

**Table 2 Final model: number of actual and predicted cases and mortalities. Results are shown for the top 21 LA with the highest predicted cases observed at wk 51 using LD_SD.**

| Local Authority | Number of Actual Cases for week 46 | Number of Actual Mortalities for week 46 | Cases Forecast for week 51 | Mortality Forecast for week 51 | Cases Forecast for week 51 | Mortality Forecast for week 51 |
|---|---|---|---|---|---|---|
| Intervention | *Full Lockdown* | | *Local lockdown with social distancing* | | *Full lockdown* | |
| Leeds | 1801 | 17 | 1881 | 21 | 499 | 17 |
| Sheffield | 948 | 36 | 1784 | 32 | 275 | 14 |
| Birmingham | 1957 | 35 | 1627 | 25 | 537 | 17 |
| Wigan | 759 | 34 | 1554 | 24 | 346 | 10 |
| Manchester | 1067 | 13 | 1550 | 20 | 427 | 13 |
| Bradford | 1534 | 24 | 1549 | 20 | 722 | 17 |
| Stockport | 517 | 13 | 1529 | 17 | 218 | 7 |
| Liverpool | 750 | 29 | 1509 | 22 | 234 | 8 |
| Rotherham | 561 | 20 | 1448 | 22 | 257 | 7 |
| Kingston upon Hull | 1011 | 21 | 1368 | 18 | 584 | 12 |
| Oldham | 735 | 15 | 1336 | 17 | 509 | 11 |
| Wirral | 311 | 13 | 1324 | 19 | 65 | 4 |
| Bolton | 635 | 18 | 1299 | 17 | 447 | 11 |
| Bristol | 763 | 8 | 1296 | 14 | 444 | 13 |
| Tameside | 447 | 18 | 1288 | 20 | 255 | 7 |

| | | | | | | |
|---|---|---|---|---|---|---|
| County Durham | 1161 | 23 | 1252 | 26 | 377 | 7 |
| Derby | 537 | 11 | 1234 | 15 | 335 | 8 |
| Walsall | 605 | 15 | 1233 | 21 | 288 | 7 |
| Kirklees | 1292 | 25 | 1207 | 29 | 557 | 14 |
| Leicester | 1006 | 7 | 993 | 15 | 581 | 11 |
| Sandwell | 762 | 21 | 969 | 25 | 398 | 10 |

LD_SD with -50% international travel was the most effective measure after full lockdown (blue vs. brown, fig 4). 100% quarantine (pink) was the next most effective supplementary measure, with similar effectiveness to international travel -50% except for three LA. Notably, LD_SD with 100% quarantine resulted in higher cases than LD_SD with international travel -50% for Bradford (+9.1%), Leicester (+7.6%). As an exception, Manchester had -41% less cases when using the quarantine measure compared to international travel restrictions.

The supplementary effect of school closing -50% was less than international travel restrictions for all 21 LA, with the number of cases being (+9.2%) higher on average using the former measure. Closing pubs -50% had a similar, albeit slightly lower level of effectiveness compared to school closing, with a higher number of cases (+2.2%) on average using the former measure compared to the latter. Again, reducing the number of food & accommodation services -50% had a similar, but slightly lower level of effectiveness compared to pubs closing, with the number of cases (+2.0%) being higher on average using the former measure. In addition, a reduction in the number of retail services -50% resulted in a similar effect to food & accommodation services -50%, with on average a minimal increase in the number of cases (+0.29%) using the former measure. It can be seen that on average, the ranking of measure effectiveness for the national hotspots are the same as the local baseline, i.e. Southampton.



For peer review only - http://bmjopen.bmj.com/site/about/guidelines.xhtml

# DISCUSSION

We have developed a deep learning model that investigates the impact of local versus national measures on COVID-19 spread in England as well as the associated mortality rates and allows forecasting based on simulation of different scenarios (http://137.222.198.54:8081/). This model can be regularly updated as the new information on actual numbers becomes available.

The temporal based deep learning model can be used to make inferences into the effectiveness of different measures at both the national and local level. The model suggests that there is variation in the effect of each measure across different regions. Notably, our results suggest that the protective effects of lockdown measures benefit some local authorities more than others (Supplementary Material, Part IV. fig S6 and S7) and that local lockdown with social distancing is ineffective compared to national lockdown in suppressing the increase in cases for most of the local authority areas. That is, if the government had kept the same local lockdown with social distancing policies, which they had implemented from week 40 onwards rather than switching to a national lockdown policy at week 45, then we would have seen a rapid rise in cases not only in the n1 belt region, but also in areas such as County Durham (n2), Bristol (s2) and Birmingham (s1), as well as in many other areas across England.

Local lockdown with social distancing may be inefficient in stopping rapid rise of hotpot regions due to geographical properties of hotspot regions. Hotspots along the middle of the n1 geographical slice constitute a tight cluster of large metropolitan cities, and the high number of cases may be partly attributed to the high number of services such as pubs and schools available as well as the amount of travel in these areas. We also expect that LA areas where there are many boundary connections to



For peer review only - http://bmjopen.bmj.com/site/about/guidelines.xhtml

other hotspots are likely to develop a higher number of cases when LD_SD measures are implemented. This is because, if one of an LA's neighbours is locked down, citizens from that LA can still travel to its other neighbours given that it has many neighbouring connections. We expect this could allow the continuation of the spread of COVID-19 within these tight cluster regions.

Since the government is only able to impose a national lockdown for a limited period, follow-up measures should improve upon LD_SD as this is likely not to be sufficient. The introduction of additional measures on top of local lockdown with social distancing can help to suppress the increase of or even decrease the number of cases in national hotspots as well as local areas where cases are not very high. Our model shows that the ranking of the average effectiveness of each supplementary measure is consistent across the national hotspots and local baseline, and this ranking can be used to prioritise those interventions according to an order of effectiveness. Nonetheless, it was also observed that certain measures are more effective for some LA compared to others. In these cases, it is necessary to adjust the priorities of the measures implemented accordingly.

The model has highlighted the importance of reducing the amount of international travel, the number of open schools and pubs as well as the implementation of travel quarantine procedures in controlling the spread of COVID-19 over other measures, such as reducing the number of food & accommodation and retail services, which seemed less relevant on the virus spread (fig 4 and fig S6). One explanation for the importance of international travel on the spread of the disease is that whilst the UK government has a certain level of control over the restriction of activities in the UK, it has little control over the activities of travelling individuals once they arrive at their destinations as well as the level of health



For peer review only - http://bmjopen.bmj.com/site/about/guidelines.xhtml

preventative measures at those destinations. Furthermore, the travelling individuals are more likely to encounter hotels, resorts, trains, planes and other places of gathering whilst abroad. These factors can contribute to the increased spread of COVID-19. Whilst travel quarantine measures provide the government to some degree the selection of which countries to enforce a 14-day self-isolation on the traveller's return, there are possible explanations why this would not be as effective as directly reducing the amount of international travel. Firstly, the restrictions do not prevent travellers (e.g. pre-university gap year students, those not working) not wary of self-isolation to travel to high risk countries. Secondly, although a penalty is imposed if self-isolation is violated, the act of self-isolation is largely dependent on the level of cooperation from the individual. Finally, even with COVID-19 testing in place, the journey from the airport back to the home of the traveller allows an increased opportunity of spreading the virus, particularly if public transport such as taxis or buses are taken.

Whilst closing schools were not as effective as international travel and quarantine restrictions, it was found to be more effective than closing pubs. One potential explanation for this is that schools are more crowded places and are subject to more frequent number of close contact scenarios in comparison to the pub. The view that schools contribute to the spread of COVID-19 have been supported by the literature.[7,8] Whilst the virus may pose low risk of mortality to the children themselves, these frequently asymptomatic carriers can also lead to the spread of the virus to their households, teachers and communities.

The reason why minimal effects were found for food & accommodation and retail restrictions may be due to the possibility that in these sectors people generally associate with others that they are closely associated with. For example, families are



For peer review only - http://bmjopen.bmj.com/site/about/guidelines.xhtml

more likely to sit with each other in restaurants or walk together with each other when shopping rather than people they are less familiar with. This is not the case in pubs as anyone from the communal area can be present.

It is unexpected that in the s2 slice that Bristol has higher predicted cases than the LA in the London area as one would have thought the latter comprising a total population of 9 million (2019) and a high traffic volume owing to its large underground network system would result in much higher case numbers. We expect that this may be because the LA in the London region generally have less health and disability deprivation (Deciles: Wandworth: 7; Barnet: 8.9; Brent: 7.3; Waltham Forest: 6.1) compared to Bristol (Decile: 4.4). This is supported by the finds which suggest that existing comorbidities are associated with an increased likelihood of COVID-19 hospital admission.[9]

In light of evidence given by the comparison between the LA within the London region and Bristol, we expect the effect of LA boundary connections to be adjusted by the degree of health and disability deprivation. Indeed, we found that regions with high number of cases along the horizontal “belt” in the n1 region had a high degree of health and disability deprivation (Decile: Manchester: 1.9; Leeds: 4.1; Bradford: 3.3; Liverpool: 1.8; Sheffield: 3.9; Wigan: 3.4). This also applies to County Durham (n2), which has a high degree of health and disability deprivation (decile: 2.9) and was seen to have a significant increase in cases at week 51 using a LD_SD measure.



For peer review only - http://bmjopen.bmj.com/site/about/guidelines.xhtml

# CONCLUSION

The present analysis found that the national lockdown was more effective than targeted local lockdown measures and its implementation from week 45 to 49 by the government was justified, albeit not sufficient to fully control the spread of COVID-19. In addition, given the limited governmental resources and timespan of the national lockdown, a rapid rise would be inevitable in health deprived and geographically vulnerable areas with a high degree of boundary connection, if only local lockdown with social distancing is used as a followed-up measure.

Our model suggests the importance of restrictions on international travel and travel quarantines, thus suggesting that follow-up policies should be comprised of the combination of local lockdown with social distancing and a reduction in the number of open airports within close proximity of the hotspot regions. Stricter measures should be placed in terms travel quarantine to increase the impact of this measure. In addition, it is recommended that where possible, education should be provided remotely, and pubs should be closed.

# FOOTNOTES

Contributors: TD and UB conceived and designed this study. TD and UB acquired the data. TD, UB, SS, and AD analysed and interpreted the data. MC and GDA provided administrative and operational support. The initial manuscript was drafted by TD; all



For peer review only - http://bmjopen.bmj.com/site/about/guidelines.xhtml

authors critically revised the manuscript and approved its final version. UB acts as guarantor. The corresponding author attests that all listed authors meet authorship criteria and that no others meeting the criteria have been omitted.

---

Copyright/license: *I, the Submitting Author has the right to grant and does grant on behalf of all authors of the Work (as defined in the below author licence), an exclusive licence and/or a non-exclusive licence for contributions from authors who are: i) UK Crown employees; ii) where BMJ has agreed a CC-BY licence shall apply, and/or iii) in accordance with the terms applicable for US Federal Government officers or employees acting as part of their official duties; on a worldwide, perpetual, irrevocable, royalty-free basis to BMJ Publishing Group Ltd ("BMJ") its licensees and where the relevant Journal is co-owned by BMJ to the co-owners of the Journal, to publish the Work in BMJ Open and any other BMJ products and to exploit all rights, as set out in our licence.*

*The Submitting Author accepts and understands that any supply made under these terms is made by BMJ to the Submitting Author unless you are acting as an employee on behalf of your employer or a postgraduate student of an affiliated institution which is paying any applicable article publishing charge ("APC") for Open Access articles. Where the Submitting Author wishes to make the Work available on an Open Access basis (and intends to pay the relevant APC), the terms of reuse of such Open Access shall be governed by a Creative Commons licence – details of these licences and which Creative Commons licence will apply to this Work are set out in our licence referred to above.*

---



For peer review only - http://bmjopen.bmj.com/site/about/guidelines.xhtml

Funding: This study was supported by the NIHR Biomedical Research Centre at University Hospitals Bristol and Weston NHS Foundation Trust and the University of Bristol. The funders had no role in the study design, data collection and analysis, decision to publish, or preparation of the manuscript. All authors had full access to all of the data (including statistical reports and tables) in the study and can take responsibility for the integrity of the data and the accuracy of the data analysis.

---

Competing interests: All authors have completed the Unified Competing Interest form (available on request from the corresponding author) and declare: no support from any organisation for the submitted work; no financial relationships with any organisations that might have an interest in the submitted work in the previous three years; no other relationships or activities that could appear to have influenced the submitted work.

---

Ethical approval: Not needed

---

Transparency: The manuscript's guarantor affirms that the manuscript is an honest, accurate, and transparent account of the study being reported; that no important



For peer review only - http://bmjopen.bmj.com/site/about/guidelines.xhtml

aspects of the study have been omitted; and that any discrepancies from the study as planned (and, if relevant, registered) have been explained.

---

Data sharing: All the data used in the study are available from public resources. The dataset is found in the Supplementary Materials document and can also be made available on request from the corresponding author.

---

## REFERENCES


1 COVID-19 cases worldwide by day. Statista. https://www.statista.com/statistics/1103040/cumulative-coronavirus-covid19-cases-number-worldwide-by-day/ (accessed 11 Dec 2020).

2 Dyer O. Covid-19: Denmark to kill 17 million minks over mutation that could undermine vaccine effort. *BMJ* 2020;**371**. doi:10.1136/bmj.m4338

3 Melin P, Monica JC, Sanchez D, *et al.* Analysis of Spatial Spread Relationships of Coronavirus (COVID-19) Pandemic in the World using Self Organizing Maps. *Chaos Solitons Fractals* 2020;**138**:109917. doi:10.1016/j.chaos.2020.109917

4 Cho K, van Merrienboer B, Bahdanau D, *et al.* On the Properties of Neural Machine Translation: Encoder-Decoder Approaches. *ArXiv14091259 Cs Stat* Published Online First: 7 October 2014.http://arxiv.org/abs/1409.1259 (accessed 10 Dec 2020).

5 Mnih V, Kavukcuoglu K, Silver D, *et al.* Human-level control through deep reinforcement learning. *Nature* 2015;**518**:529–33. doi:10.1038/nature14236

6 Zou F, Shen L, Jie Z, *et al.* A Sufficient Condition for Convergences of Adam and RMSProp. In: *2019 IEEE/CVF Conference on Computer Vision and Pattern Recognition (CVPR)*. Long Beach, CA, USA: : IEEE 2019. 11119–27. doi:10.1109/CVPR.2019.01138

7 Sheikh A, Sheikh A, Sheikh Z, *et al.* Reopening schools after the COVID-19 lockdown. *J Glob Health*;**10**. doi:10.7189/jogh.10.010376




For peer review only - http://bmjopen.bmj.com/site/about/guidelines.xhtml

8 Stein-Zamir C, Abramson N, Shoob H, *et al.* A large COVID-19 outbreak in a high school 10 days after schools' reopening, Israel, May 2020. *Eurosurveillance* 2020;**25**:2001352. doi:10.2807/1560-7917.ES.2020.25.29.2001352

9 Price-Haywood EG, Burton J, Fort D, *et al.* Hospitalization and Mortality among Black Patients and White Patients with Covid-19. *N Engl J Med* 2020;**382**:2534–43. doi:10.1056/NEJMsa2011686

For peer review only

## LEGENDS

Fig 1 Validation of cases for week 46 with weeks 41 to 46 excluded from data

Fig 2 Geographical level of cases for actual and predicted results based on different measures. a) exemplifies the use of geographical slices n2, n1, s1, s2. Additional results are available in Supplementary Materials, Part III.

Fig 3 For the top 21 LA with the highest predicted cases observed at wk 51 using LD_SD, plots were generated to compare the effects of full lockdown against LD_SD in terms of cases a) and mortalities b).

Fig 4 For the top 21 LA with the highest predicted cases observed at wk 51 using LD_SD, a plot is generated to compare the effect on the number of cases using a combination of LD_SD with other "supplementary" measures.



For peer review only - http://bmjopen.bmj.com/site/about/guidelines.xhtml

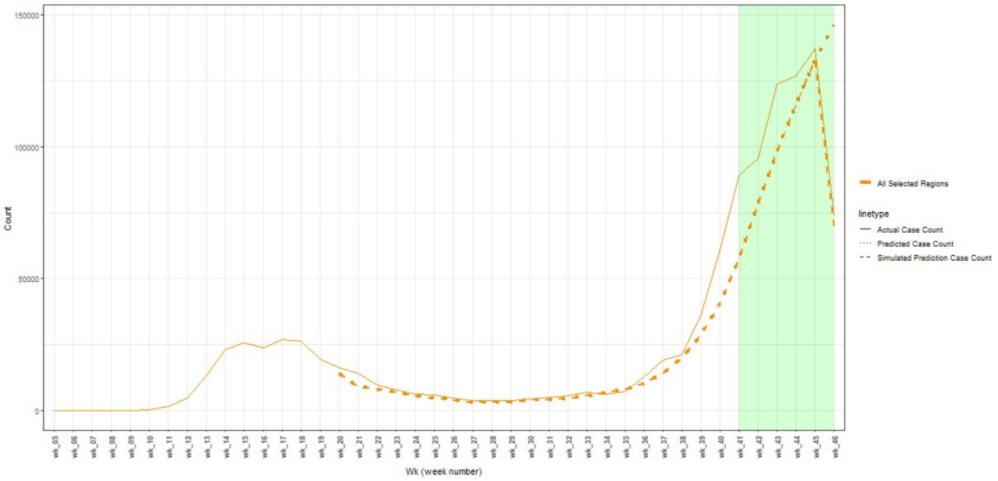


Fig 1 Validation of cases for week 46 with weeks 41 to 46 excluded from data

141x68mm (300 x 300 DPI)

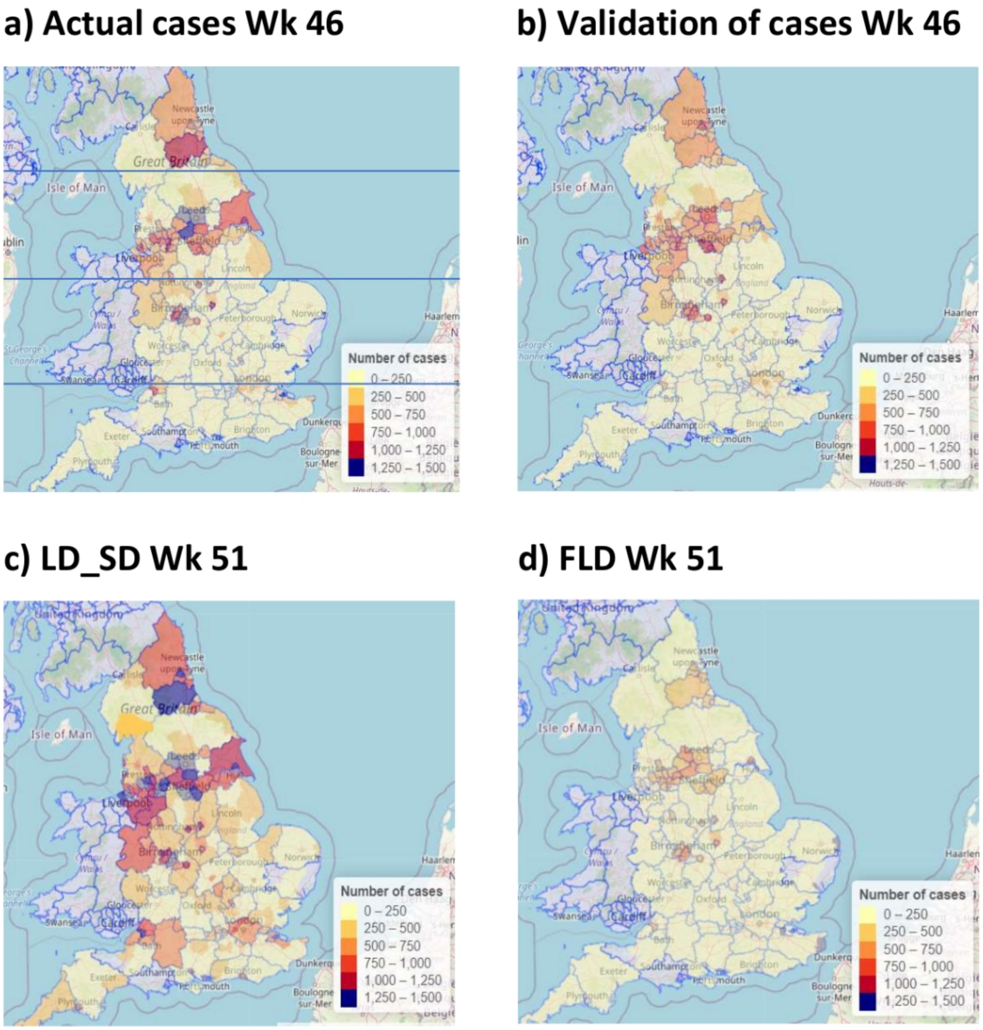


Fig 2 Geographical level of cases for actual and predicted results based on different measures. a) exemplifies the use of geographical slices n2, n1, s1, s2. Additional results are available in Supplementary Materials, Part II. B.

102x106mm (300 x 300 DPI)

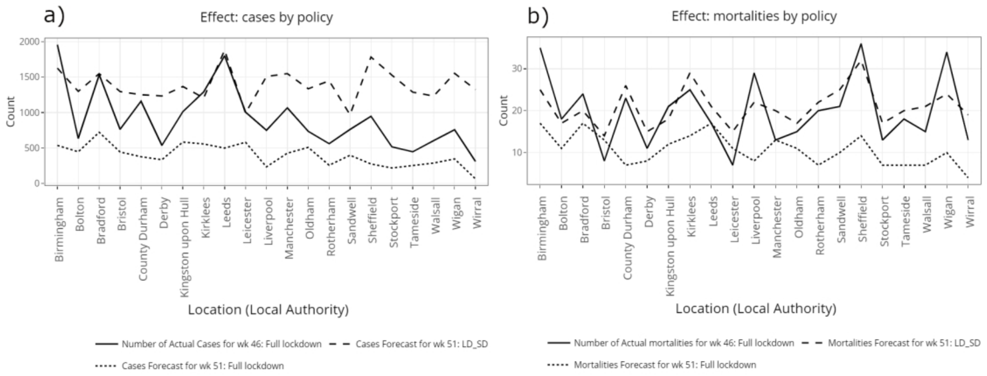


Fig 3 For the top 21 LA with the highest predicted cases observed at wk 51 using LD_SD, plots were generated to compare the effects of full lockdown against LD_SD in terms of cases a) and mortalities b).

151x57mm (300 x 300 DPI)

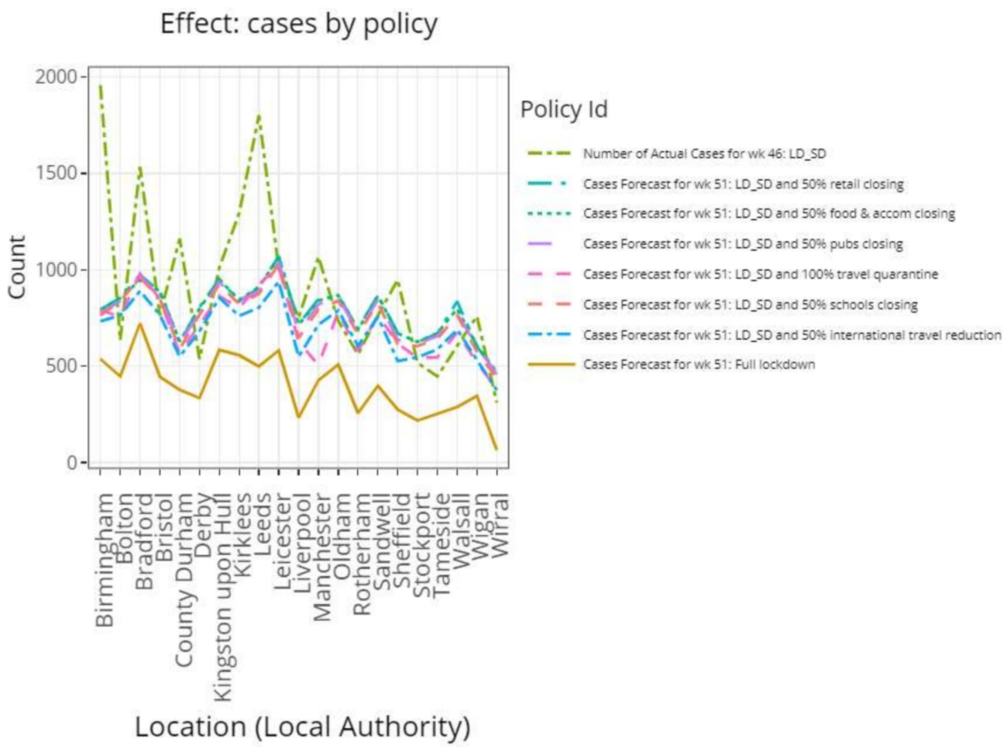


Fig 4 For the top 21 LA with the highest predicted cases observed at wk 51 using LD_SD, a plot is generated to compare the effect on the number of cases using a combination of LD_SD with other "supplementary" measures.

155x115mm (300 x 300 DPI)

# Supplementary Materials: A Deep Recurrent Reinforced Learning model to compare the efficacy of targeted local vs national measures on the spread of COVID-19 in the UK

*Tim Dong (0000-0003-1953-0063), Umberto Benedetto (0000-0002-7074-7949), Shubhra Sinha, Arnaldo Dimagli, Massimo Caputo, Gianni Angelini*

Part I.

## GRU Gated Recurrent Units Model

We have chosen a Recurrent Neuro Network (RNN) category of model because of the ability of this type of model to model not only non-linear relationships between high dimension of variables, but also because of its ability to model temporal relationships within any of the variables considered. As figure S1. shows, the RNN model consists of cells is analogous to each unit of the traditional neuro network. Whilst traditional neuro network models accumulate weights $W_i$ (input to hidden layer) and $W_h$ (hidden to hidden layer), they do not calculate any recurrent weights $W_r$ that represent temporal relationships of each variable in the dataset. This recurrent type of weight is present in RNN models and hence is desirable for modelling a time dependent COVID-19 dataset.

For peer review only

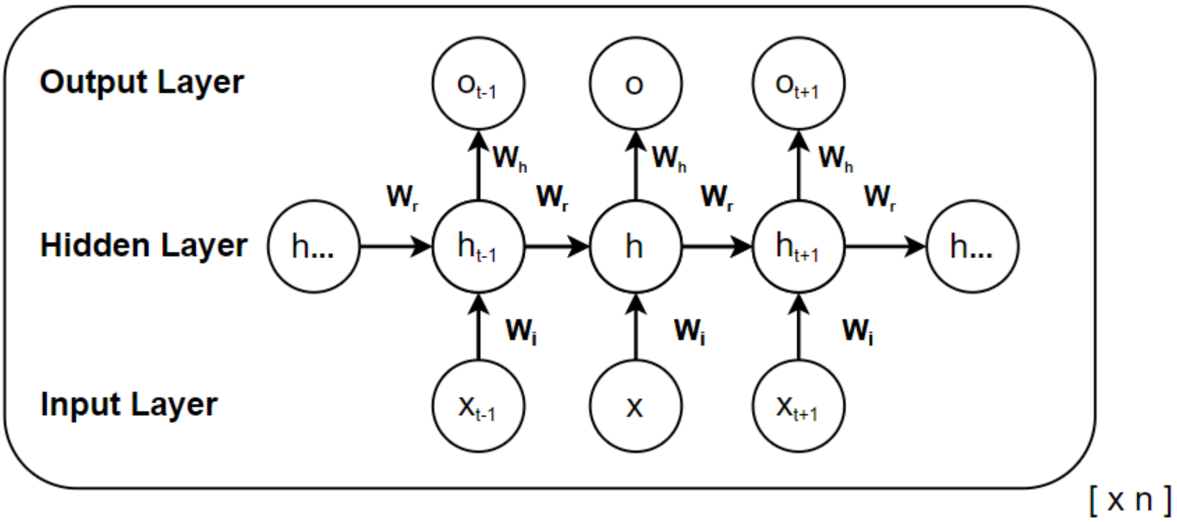


Figure S1. An RNN cell

GRU is a specific type of Recurrent Neuro Network (RNN) that incorporates long term memory and effectively deals with the vanishing gradient and gradient explosion problems that have affected various other categories of RNN. The GRU model was also selected because of its ease to optimise and its low computational cost in comparison to the LSTM model.

Unlike the LSTM model, which uses four gates, the GRU model uses only a single decision gate that controls both the update and reset of weights. The update gate is similar to an OR gate in electronics that either retains the state at the previous step $h_{t-1}$ or updates the previous state based on variable values $X_t$ provided at the current step. The reset gate is much like a resistor in that it controls that size of the effect from previous step that contributes to the current step.

For peer review only

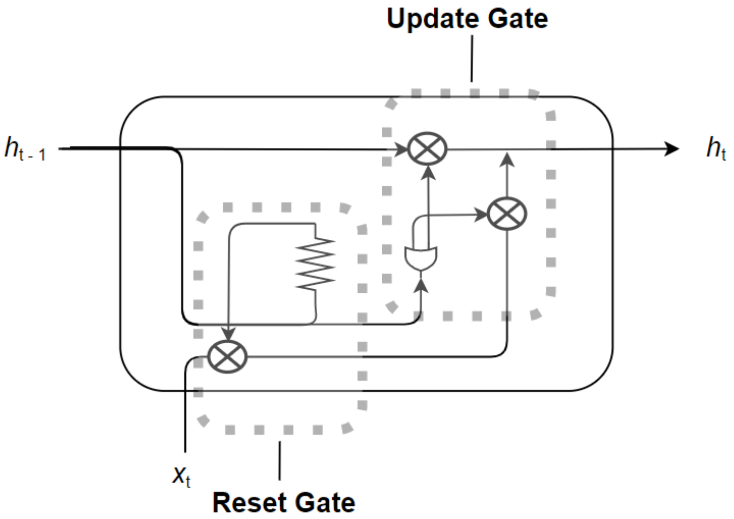


Figure S2. A GRU cell

The above diagram above provides an intuitive understanding into the processes that occur within a cell or node of a GRU model. The specific mathematical equation of the GRU model is given below:

$$h_t^i = z_t^i . h_{t-1}^i + \left(1 - z_t^i\right) tanh\left(b^i + \sum_j W_I^{i,j} x_t^j + \sum_j W_R^{i,j} r_{t-1}^j h_{t-1}^j\right)$$

$$z_t^i = \sigma\left(b_z^i + \sum_j W_z^{i,j} x_t^j + \sum_j W_z^{i,j} h_{t-1}^j\right)$$

$$r_t^j = \sigma\left(b_r^i + \sum_j W_r^{i,j} x_t^j + \sum_j W_r^{i,j} h_{t-1}^j\right)$$

where z represents for the update gate and r represents the reset gate, $W_I$ are the weights from the input to hidden layer, $W_R$ are the weights from the recurrent weights, b is the bias, $\sigma$ and tanh are the sigmoid and tanh activation functions respectively, $X_t$ are the inputs at time t and $h_{t-1}$ was the input from previous time step.

**Deep Reinforcement Learning**

Deep reinforcement learning is the application of reinforcement learning to neuro networks. The application of this approach has been exemplified in the field of computer gaming.[1] Essentially, the approach involves an agent that represents the computer system, which performs an action in the environment that it interacts with.

For peer review only - http://bmjopen.bmj.com/site/about/guidelines.xhtml

The environment is changed following the action and a reward is provided to the agent such that the action it selects from a list of actions or policy $\pi$ is optimised in any subsequent interactions with the environment.

Here, we present a technique whereby the NCGFS model represents the agent. It is initially presented with a set of observations $O_t$ from the national environment, *s*. Each individual observation has the property $o \in A$, where $A$ is the set of all the LA. Rather than using the traditional reward variable *r*, we use $L_t$ to represent the loss at the initial training phase *t* of the deep learning model, i.e. at the generation point of model-M. Subsequently, when the agent is presented with the input observations $O_{t+1}$ that belongs to a single local authority, $s_{t+1}$ from the set A, it uses the action or in this case forecast from the list of potential forecasts that previously minimised the $L_t$ for $O_t$ to make the forecast $f_t$ for $O_{t+1}$. This induces a loss $L_{t+1}$ that is used to update the NCGFS model's policy decisions for the actual prediction of $O_{t+1}$ as $f_{t+1}$. The resulting model is termed model-R.

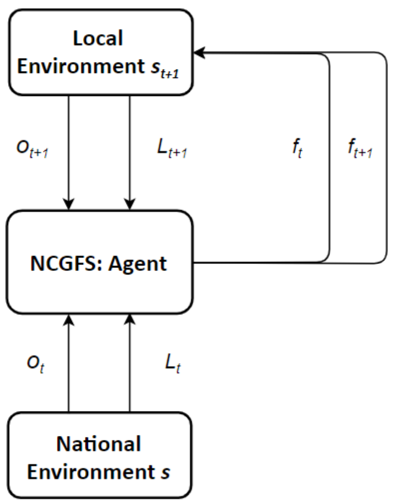


Figure S3. illustration of the Deep Reinforcement Learning aspect of NCGFS at the local authority level.

The following equation shows how NCGFS uses deep reinforcement learning to minimise the Loss $L$ for a given environment s to produce an optimal forecast from the probabilistic distribution of forecasts $\pi = P(f|s)$.

$$Q^{*}(s,\ f) \ = \min_{\pi} \ \frac{1}{n}\left(\sum_{i}^{n} \gamma^{i} L_{t+i} \mid s_t \ = \ s,\ f_t \ = \ f,\ \pi\right)$$

Neural Network architecture and configuration

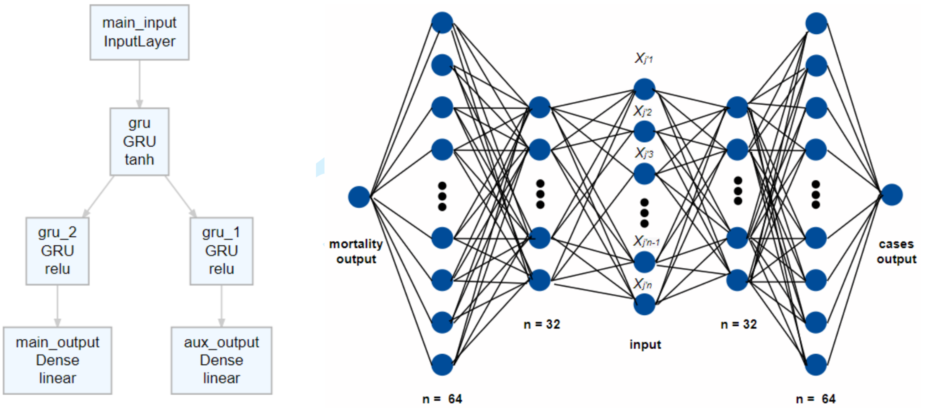


Figure S4.1 NCGFS model architecture. Note, the two layers on either side of the input layer have been depicted in this manner to facilitate representation but represent the same layer.

The model is comprised of symmetrical neuro network that consists of six layers. The input layer accepts a data matrix in the dimension of $[d \quad \tau \quad n]$, where $d$ = 136 is the number of variables, $\tau$ = 2 is the number of time steps in the recurrent direction along the hidden layer, $n$ is the number of samples taken from the observation. The layer immediately right of the input layer is named gru and consists of 32 GRU cells or units. The next layer to the right is name gru_2 and consists of 64 GRU cells. The output from this layer uses the ReLU activation function as this has been demonstrated to be effective for the convergence of neuro networks. This is followed by a dense output layer named main_output, consisting of one unit on the right. This

layer provides the prediction output for the number of COVID-19 cases five weeks ahead of the corresponding weeks in the input data.

The layer immediately left of the input layer is in fact the same layer as that to the right of input layer i.e. named gru. The next layer to the left is a named gru_1 and consists of 64 GRU cells. This is followed by a dense output layer named aux_output for prediction the number of mortalities five weeks ahead of the corresponding weeks in the input data.

We have empirically found that this combination of depth and width is efficient for the minimising the loss of the learning problem at hand.

Model-M was trained using four iterations of 500 epochs (with 6 steps per training epoch and 1 step per validation epoch) using early stop to stop training once validation loss has stopped decreasing with a difference of 0.001 and patience of 200. With the four iterations of training, NCGFS model-M had achieved a performance validation loss of 0.17456 consisting of the sum of validation loss for both cases and mortality.

Part II.

1. File 2: domains of deprivation

url location:

https://www.gov.uk/government/statistics/english-indices-of-deprivation-2019

2019 indices containing individual metrics including health, education

Index of Multiple Deprivation (IMD) Decile (where 1 is most deprived 10% of LSOAs)

IMD Rank (1 is most deprived)

2. 2001 to 2018 edition of this dataset

For peer review only

Public houses and bars by local authority

url location:

https://www.ons.gov.uk/businessindustryandtrade/business/activitysizeandlocation/datasets/publichousesandbarsbylocalauthority

Using Pubs size LA 2018 by local authority. Gives the number of pubs in UK by local authority

3. 2020 edition of this dataset

UK business: activity, size and location

url location:

https://www.ons.gov.uk/businessindustryandtrade/business/activitysizeandlocation/datasets/ukbusinessactivitysizeandlocation

ukbusinessworkbook2020.csv by Retail, Transport_Storage_inc_postal, Accommodation_food services, Education, Health, Arts_entertainment recreation_other_services

4. Local Authority Districts 2019 boundaries

Local Authority Districts in the United Kingdom, as at 31 December 2019. The boundaries available are:

- (BUC) Ultra Generalised (500m) - clipped to the coastline (Mean High Water mark).

https://geoportal.statistics.gov.uk/datasets/local-authority-districts-december-2019-boundaries-uk-buc

We use this as COVID-19 cases data is based on 2019 LA boundaries

5. Local Authority District to Public Health England Centre to Public Health England Region (December 2019) Lookup in England

url:

https://geoportal.statistics.gov.uk/datasets/local-authority-district-to-public-health-england-centre-to-public-health-england-region-december-2019-lookup-in-england

For peer review only

6. Mid-2019: April 2019 local authority district codes edition of this dataset

Population estimate data

url:
https://www.ons.gov.uk/peoplepopulationandcommunity/populationandmigration/populationestimates/datasets/populationestimatesforukenglandandwalesscotlandandnorthernireland

Using the following sheets: MYE2 – Males, MYE2 – Females, MYE3 (migrationFlow), MYE2 – Persons (contains age population).

7. SchoolOpening

url:

https://www.gov.uk/government/publications/actions-for-schools-during-the-coronavirus-outbreak/guidance-for-full-opening-schools

https://www.bbc.co.uk/news/uk-51952314

Schools have remained open to some pupils since 23 March, welcoming more pupils back from 1 June.

During model generation, the SchoolOpening index was linked to the main dataset based on the weeks the school restriction policies were implemented and the relative effects at each time period. Index definition: 3 = No restrictions; 1.5 = School closing but remaining open to some pupils; 3 = Also used to represent school reopening.

Unlike the LockdownScore indices, the SchoolOpening index already takes into account of the effect that school closing typically occurs when the number of cases or mortality is highest, by inversing scoring. Hence, in this case when varying the SchoolOpening parameter during model configuration mode, this metric will not have an inverse effect.

Note, the effects pertain only to the local authorities selected.

For peer review only

8. Number of monthly arrivals in tourist accommodation in Spain from August 2018 to July 2020*

url:

[https://www.statista.com/statistics/1130775/number-of-monthly-arrivals-short-stay-accommodation-in-spain/](https://www.statista.com/statistics/1130775/number-of-monthly-arrivals-short-stay-accommodation-in-spain/)

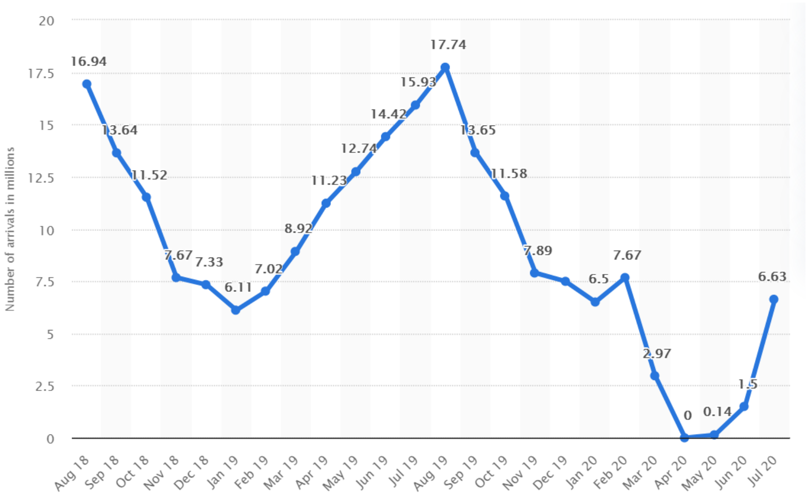


Figure S4.2. Monthly worldwide tourist arrivals in accommodation in Spain

We adjust tourist arrival by the proportion of UK citizens travelling to Spain in 2019

(section 9.)

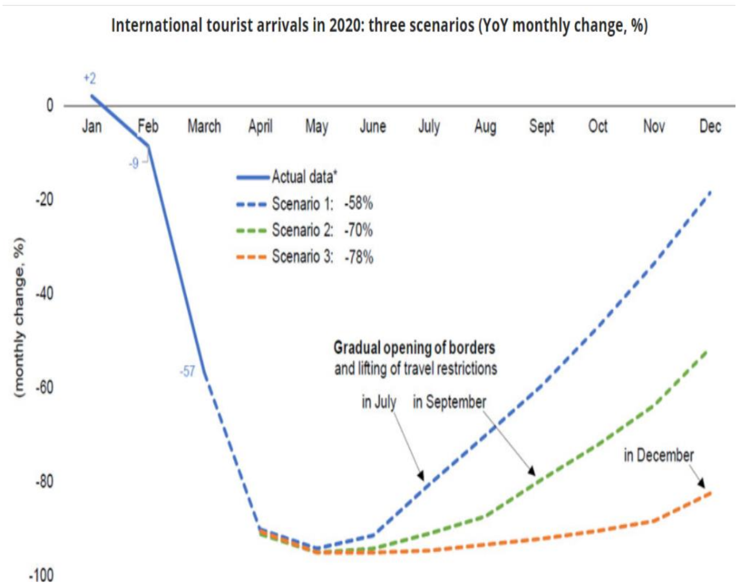


Figure S4.3 A reference model for understanding how quarantine measures are modelled.

url: https://www.adpr.co.uk/blog/covid-19/travel-and-tourism-brands-can-recover-from-coronavirus/

As UK removed 75 countries from quarantine list in July, we impute missing values from August to October based on the recovery model for July opening of borders above. However, the actual recovery based on the data is much faster than that shown in figure above.

9. Number of international tourists arriving in Spain in 2019, by country of residence

url:
https://www.statista.com/statistics/447683/foreign-tourists-visiting-spain-by-country-of-residence/

Obtains proportion of UK citizens arriving in Spain in 2019 as estimate for proportion in 2020

statistic_id447683_international-tourist-arrivals-in-spain-2019-by-country-of-residence.xlsx

UK proportion: 0.215984348

This is the estimated number of tourists arriving in Spain from UK in millions. The values are calculated based on number of tourists from various countries arriving in Spain from Jan 2020 to July 2020 and then adjusting by multiplying this number by the proportion of UK tourists in Spain from the preceding year i.e. 2019.

The weeks not covered by the data available i.e. August to October are imputed based on the tourism industry recovery model for July opening of borders https://www.adpr.co.uk/blog/covid-19/travel-and-tourism-brands-can-recover-from-coronavirus/.

This metric should be used as a guidance for how the situation will vary when international travel is restricted.

For peer review only

10. Public Health England Centres (December 2016) Ultra Generalised Clipped Boundaries in England

url:

https://geoportal.statistics.gov.uk/datasets/public-health-england-centres-december-2016-ultra-generalised-clipped-boundaries-in-england

2016 is the latest available

11. QuarantineMeasures

url:

https://webcache.googleusercontent.com/search?q=cache:J8RTskJUc0QJ:https://www.theweek.co.uk/107044/UK-coronavirus-timeline+&cd=4&hl=en&ct=clnk&gl=uk

During model generation, the QuarantineMeasures index were linked to the main dataset based on the weeks the travel quarantine policies were implemented and the relative effects at each time period. Index definition: 1 = No quarantine; 10 = full quarantine of tourist from all countries; 5 = Removal of 59 countries from quarantine list; 5.5 = Adding Spain back to the quarantine list following removal of 59 countries from the list.

As the level of travel quarantine restrictions implemented is dependent on the severity of covid-19 situation in other countries rather than the number of cases and mortality in UK, when varying this parameter during model configuration mode, this metric will have not an inverse effect.

Note, the effects pertain only to the local authorities selected.

12. LockdownScore

For peer review only

During model generation, the LockdownScore index were linked to the main dataset based on the weeks the lockdown policies were implemented and the relative effects at each time period. Index definition: 1 = No lock down; 4 = Local lock down; 5 = Local lock down with social distancing; 10 = Full Lock Down.

However, as the level of lock down implemented is typically highest when the number of cases or mortality is highest, when varying this parameter during model configuration mode, this metric will have an inverse effect. That is, changing the LockdownScore value to 1 will result in a full lock down, whilst changing to 10 will result in no lock down. 4 will result in Local lock down with social distancing and 5 will represent Local lock down.

Note, the effects pertain only to the local authorities selected.

Part III.

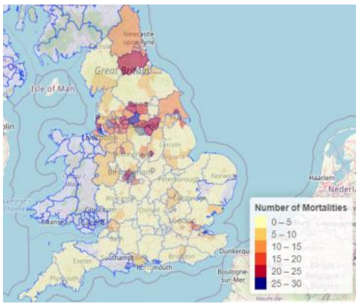


(i) M: Actual Mortalities Wk 46

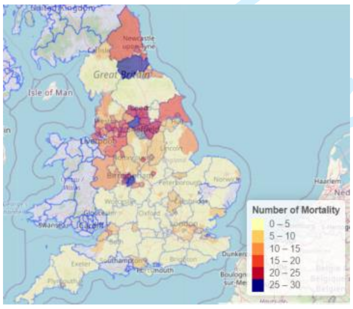


(ii) M: Wk 51 Local LD_SD

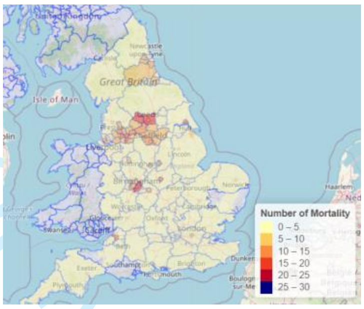


(iii) M: Wk 51 FLD

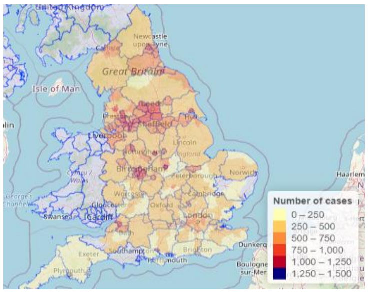


(iv) C: LD_SD -50% school

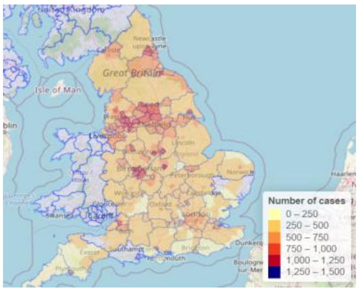


(v) C: LD_SD -50% food & accom

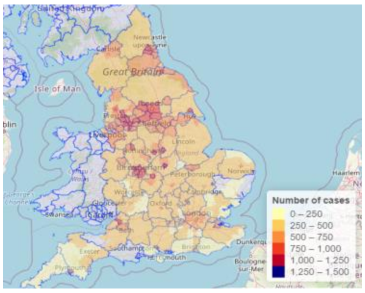


(vi) C: LD_SD -50% retail

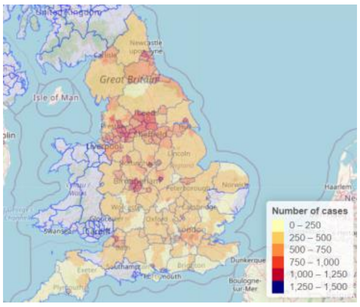


(vii) C: LD_SD -50% pubs

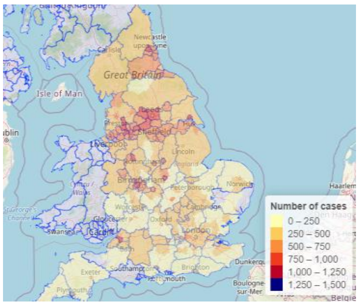


(viii) C: LD_SD intl travel -50%

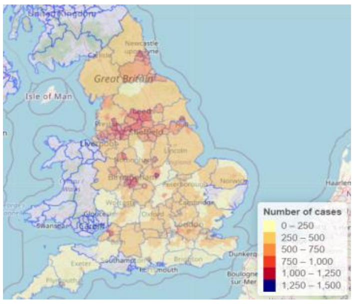


(ix) C: LD_SD 100% quarantine

Figure S5. Geographical level of cases and mortality for actual and predicted results based on different measures. C: cases; M: mortalities.

Part IV.

The deep learning model was used to generate the plots for fig S5. of the effects on the predicted number of cases for Southampton based on different measures.

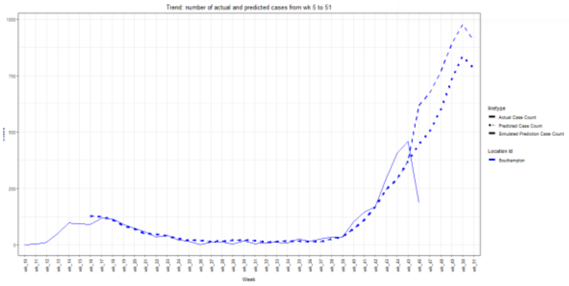


(a) No lockdown vs. LD_SD

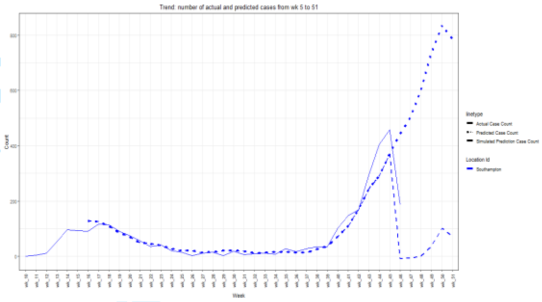


(b) LD_SD vs. full lockdown

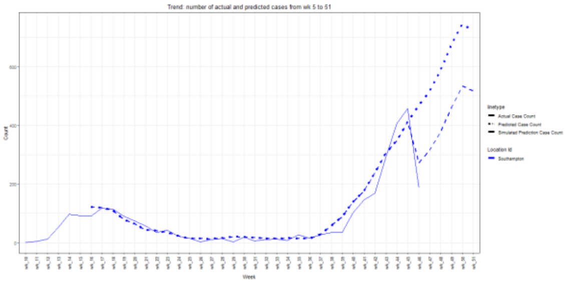


(c) LD_SD vs. international travel -50%

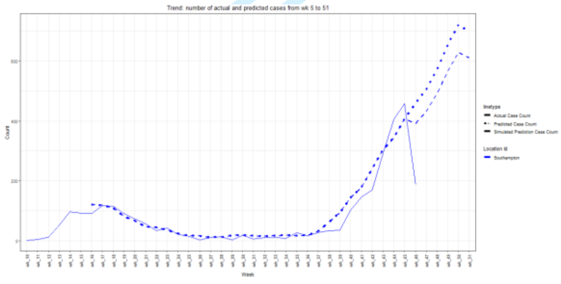


(d) LD_SD vs. closing school -50%

For peer review only

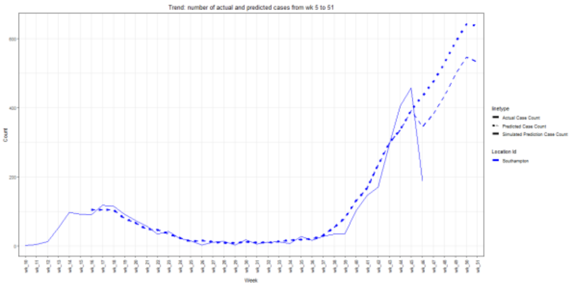

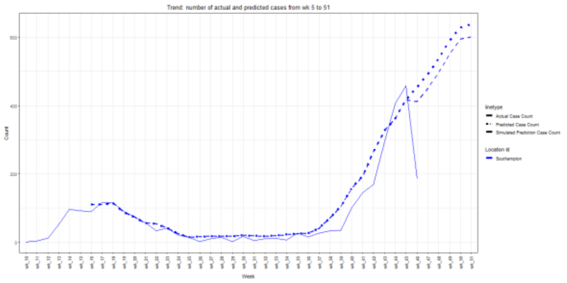

(e) LD_SD (quarantine 5.5) vs. full quarantine (10) (f) LD_SD (100% pubs) vs. -50% pubs

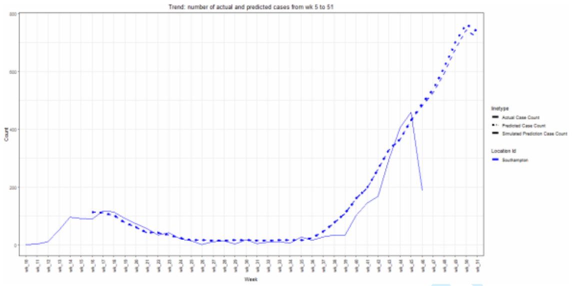

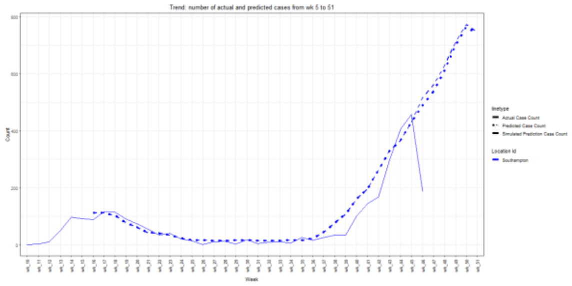

(g) LD_SD (100% food & Accom) vs. -50% (h) LD_SD (100% Retail) vs. -50% Retail

Figure S6. Cases forecast for Southampton by measure. The dotted line represents predictions using the LD_SD measures. The dashed lines represent prediction changes based on changes to measures. (c-h) relate to LD_SD without (left) and with (right) the supplementary measures.

For peer review only

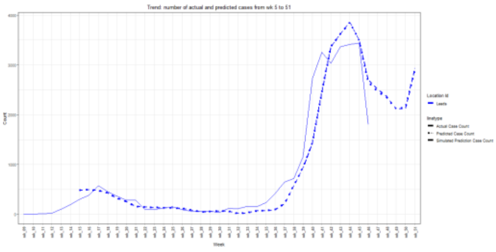

(a) No lockdown vs. LD_SD

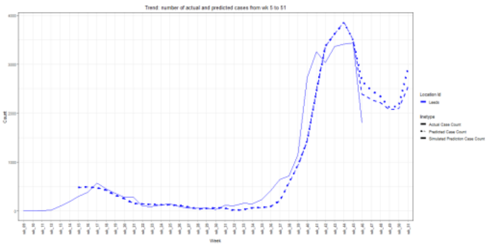

(b) LD_SD vs. full lockdown

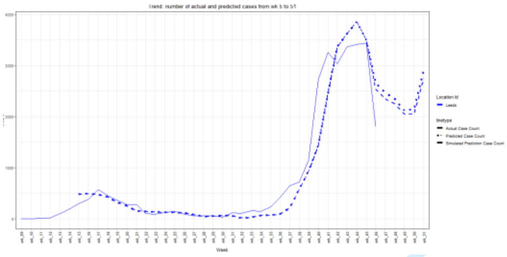

(c) LD_SD vs. international travel -50%

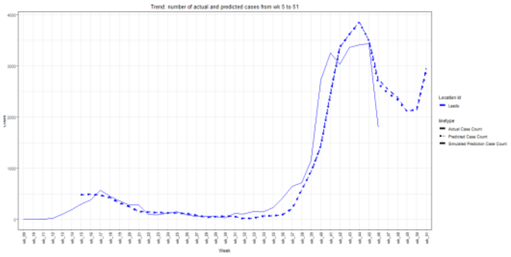

(d) LD_SD vs. closing school -50%

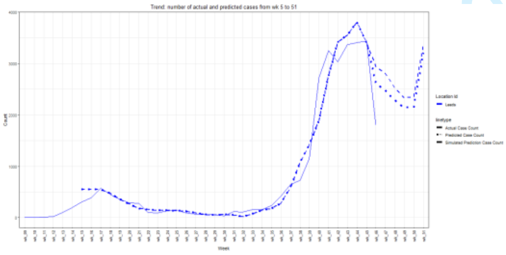

(e) LD_SD (quarantine 5.5) vs. full quarantine (10)

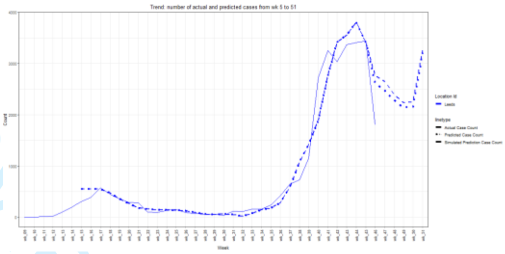

(f) LD_SD (100% pubs) vs. -50% pubs

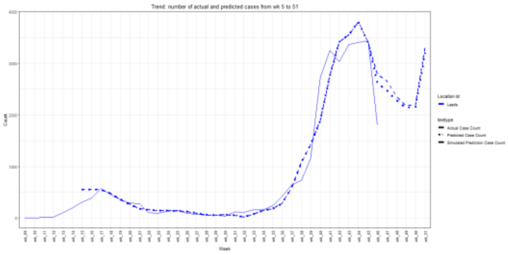

(g) LD_SD (100% food & Accom) vs. -50%

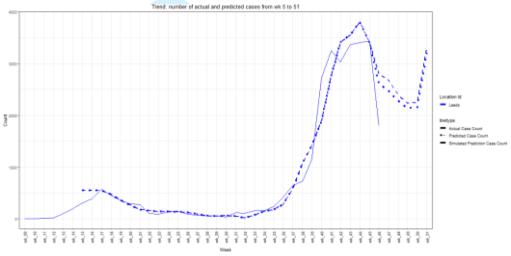

(h) LD_SD (100% Retail) vs. -50% Retail

Figure S7. Cases forecast for Leeds by measures. The dotted line represents predictions using the LD_SD measures. The dashed lines represent prediction changes based on changes to measures. (c-h) relate to LD_SD without (left) and with (right) the supplementary measures.

## References

1 Mnih V, Kavukcuoglu K, Silver D, *et al.* Human-level control through deep reinforcement learning. *Nature* 2015;**518**:529–33. doi:10.1038/nature14236

For peer review only

BMJ Open

# A Deep Recurrent Reinforced Learning model to compare the efficacy of targeted local vs. national measures on the spread of COVID-19 in the UK

| | |
|---|---|
| Journal: | *BMJ Open* |
| Manuscript ID | bmjopen-2020-048279.R1 |
| Article Type: | Original research |
| Date Submitted by the Author: | 02-Dec-2021 |
| Complete List of Authors: | Dong, Tim; University of Bristol, Bristol Heart Institute, Bristol Medical School<br>Benedetto, Umberto; University of Bristol, Bristol Heart Institute, Bristol Medical School<br>Sinha, Shubhra ; University of Bristol, Bristol Heart Institute, Bristol Medical School<br>Fudulu, Daniel; University of Bristol,  Bristol Heart Institute, Bristol Medical School<br>Dimagli, Arnaldo ; University of Bristol, Bristol Heart Institute, Bristol Medical School<br>Chan, Jeremy; University of Bristol, Bristol Heart Institute, Bristol Medical School<br>Caputo, Massimo; University of Bristol, Bristol Heart Institute, Bristol Medical School<br>Angelini, Gianni; University of Bristol, Bristol Heart Institute, Bristol Medical School |
| <b>Primary Subject Heading</b>: | Epidemiology |
| Secondary Subject Heading: | Infectious diseases, Health informatics, Public health, Research methods |
| Keywords: | COVID-19, VIROLOGY, Infection control < INFECTIOUS DISEASES, Health informatics < BIOTECHNOLOGY & BIOINFORMATICS, Epidemiology < INFECTIOUS DISEASES, PUBLIC HEALTH |
| | |

SCHOLARONE™
Manuscripts

*I, the Submitting Author has the right to grant and does grant on behalf of all authors of the Work (as defined in the below author licence), an exclusive licence and/or a non-exclusive licence for contributions from authors who are: i) UK Crown employees; ii) where BMJ has agreed a CC-BY licence shall apply, and/or iii) in accordance with the terms applicable for US Federal Government officers or employees acting as part of their official duties; on a worldwide, perpetual, irrevocable, royalty-free basis to BMJ Publishing Group Ltd ("BMJ") its licensees and where the relevant Journal is co-owned by BMJ to the co-owners of the Journal, to publish the Work in this journal and any other BMJ products and to exploit all rights, as set out in our licence.*

*The Submitting Author accepts and understands that any supply made under these terms is made by BMJ to the Submitting Author unless you are acting as an employee on behalf of your employer or a postgraduate student of an affiliated institution which is paying any applicable article publishing charge ("APC") for Open Access articles. Where the Submitting Author wishes to make the Work available on an Open Access basis (and intends to pay the relevant APC), the terms of reuse of such Open Access shall be governed by a Creative Commons licence – details of these licences and which Creative Commons licence will apply to this Work are set out in our licence referred to above.*

*Other than as permitted in any relevant BMJ Author's Self Archiving Policies, I confirm this Work has not been accepted for publication elsewhere, is not being considered for publication elsewhere and does not duplicate material already published. I confirm all authors consent to publication of this Work and authorise the granting of this licence.*

For peer review only

# A Deep Recurrent Reinforced Learning model to compare the efficacy of targeted local vs. national measures on the spread of COVID-19 in the UK

*Tim Dong[1†] (0000-0003-1953-0063), Umberto Benedetto (0000-0002-7074-7949)[2†*], Shubhra Sinha[3], Daniel Fudulu[4], Arnaldo Dimagli[5], Jeremy Chan[6], Massimo Caputo[7], Gianni D Angelini[8],*

[1] Assistant Database Manager, Bristol Heart Institute, Bristol Medical School, University of Bristol BS2 8DZ, United Kingdom

[2] Professor in Cardiac Surgery, Bristol Heart Institute, Bristol Medical School, University of Bristol BS2 8DZ, United Kingdom

[3] National Trainee Number in Cardiothoracic Surgery, Bristol Heart Institute, Bristol Medical School, University of Bristol BS2 8DZ, United Kingdom

[4] NIHR Clinical Lecturer, Bristol Heart Institute, Bristol Medical School, University of Bristol BS2 8DZ, United Kingdom

[5] Assistant Database Manager, Bristol Heart Institute, Bristol Medical School, University of Bristol BS2 8DZ, United Kingdom

[6] National Trainee Number in Cardiothoracic Surgery, Bristol Heart Institute, Bristol Medical School, University of Bristol BS2 8DZ, United Kingdom

[7] British Heart Foundation Professor of Congenital Heart Surgery, Bristol Heart Institute, Bristol Medical School, University of Bristol BS2 8DZ, United Kingdom



For peer review only - http://bmjopen.bmj.com/site/about/guidelines.xhtml

[8] British Heart Foundation Professor of Cardiac Surgery, Bristol Heart Institute, Bristol Medical School, University of Bristol BS2 8DZ, United Kingdom

[*] Corresponding Author; umberto.benedetto@bristol.ac.uk; +44 (0) 117 3428854

[†] TD and UB contributed equally to this paper

# ABSTRACT

**Objectives** To prevent the emergence of new waves of COVID-19 caseload and associated mortalities, it is imperative to understand better the efficacy of various control measures on the national and local development of this pandemic in space–time, characterise hotspot regions of high risk, quantify the impact of under-reported measures such as international travel and project the likely effect of control measures in the coming weeks.

**Methods** We applied a Deep Recurrent Reinforced Learning (DRRL) based model to evaluate and predict the spatiotemporal effect of a combination of control measures on COVID-19 cases and mortality at the local authority (LA) and national scale in England, using data from week (wk) 5 to 46 of 2020, including an expert curated control measure matrix, official statistics/government data and a secure web dashboard to vary magnitude of control measures.

**Results** Model predictions of the number of cases and mortality of COVID-19 in the upcoming five weeks closely matched the actual values (Cases: RMSE 700.88, MAE 453.05, MAPE 0.46, Correlation Coefficient 0.42; Mortality: RMSE 14.91, MAE 10.05, MAPE 0.39, Correlation Coefficient 0.68). Local lockdown with social distancing (LD_SD) (Overall Rank 3) was found to be ineffective in preventing outbreak rebound following lockdown easing compared to national lockdown (Overall

For peer review only

Rank 2), based on prediction using simulated control measures. The ranking of the effectiveness of adjunctive measures for LD_SD were found to be consistent across hotspot and non-hotspot regions. Adjunctive measures found to be most effective were international travel and quarantine restrictions.

**Conclusions** This study highlights the importance of using adjunctive measures in addition to LD_SD following lockdown easing and suggests the potential importance of controlling international travel and applying travel quarantines. Further work is required to assess the effect of variant strains and vaccination measures.

**Strengths and limitations of this study**

- The proposed Deep Recurrent Reinforced Learning (DRRL)-based model takes into account of both relationships of variables across local authorities and across time, using ideas from reinforcement learning to improve predictions.
- Whilst, predicting the geographical trend in COVID-19 cases based on the simulation of different measures in the UK at both the national and local levels in the UK has proved challenging, this study has provided a methodology by which useful predictions and simulations can be obtained.
- The Office for National Statistics only released data on UK international travel up to March 2019 at the time of this study, and therefore this study used the amount of UK tourists in Spain as a reference variable for understanding the effect of international travel on COVID-19 spread.



For peer review only - http://bmjopen.bmj.com/site/about/guidelines.xhtml

## INTRODUCTION

COVID-19 is a highly infectious disease that resulted in a global pandemic in just under a month. This pandemic has caused global disruptions to individuals, businesses and governments worldwide. The number of cases have continued to rise exponentially, from 80,239 in February 2020 to 69 million as of December 2020 a month.[1] This pandemic has caused global disruptions to individuals, businesses and governments worldwide. The number of cases has continued to rise exponentially, from 80,239 in February 2020 to 69 million as of December 2020.[1] COVID-19 is unlike other historic pandemics in terms of its rapid worldwide spread, a substantial increase in infected and symptomatic people, and a rapid development of newly evolving strains. Recent cases of a new variant of COVID-19 have also been found [2]. These problems are being faced worldwide despite global efforts to control this virus.

The spread of COVID-19 can be modelled as a four-stage process: 1) Appearance of disease; 2) Local transmission; 3) Community transmission; 4) Epidemic outbreak.[3] An area can be defined as a liberal zone, a surveillance zone, or an infected zone, depending on regional infection patterns, and different levels of restriction measures can be applied.[3] Studies have also focused on the effect of quarantine on COVID-19 spread and have found that it is more effective than control and combinations with other measures e.g. school closures, travel restrictions, and social distancing had a synergistic effect.[4]

Epidemic models developed so far have aimed at understanding the effect of various quarantine factors and mostly applied Newtonian calculus approaches.[5]



For peer review only - http://bmjopen.bmj.com/site/about/guidelines.xhtml

One study modelled the strictness of lockdown interventions using a contact factor F (ranging from 3 to 8), with three being the strongest and eight being the weakest.[6] In another study, researchers used a COVID-19 decision-making system (CDMS) based on differential formulas and stochastic methods to model transitions between population phase states such as susceptible, exposed, infected, hospitalised, recovered, and died. The study was extended to incorporate demographics and social status variables using data from official statistics and the literature.[7] In time-series data analysis and forecasting, Deep Learning (DL) shows promise. DL models can automatically learn temporal connections and patterns in the data, such as trends and seasonality.[8] Time series and geographical data analysis have been applied to study and inform on optimal energy sector management policies to mitigate the effect of COVID-19.[9] Another study also visualised the geographical distribution of COVID-19 cases.[10] For forecasting worldwide COVID-19 incidence as well as for country- and city-specific predictions, one study employed statistics measures to sort the most effective model for medium-term prediction utilising ARIMA, LSTM, Stacked LSTM (SLSTM), and Prophet models.[11] NAR and FITNET neural networks were combined as an ensemble using a fuzzy weighted approach to predict 10 days ahead of 12 Mexican states.[12] Fuzzy rules have been applied, along with Fractal Dimension as transformation criteria, to account for linear and non-linear dimensionality in order to forecast the COVID-19 trend.[13] Following this approach, expert knowledge was used to define rules and class memberships with a different set of countries.[14] To model the effect of control measures, a control loop system was utilised with a novel set of fuzzy logic, with the error between the observed and desired number of infections and the linear fractal dimension of the country as input.[15]



For peer review only - http://bmjopen.bmj.com/site/about/guidelines.xhtml

# RESEARCH GAP

From the literature review, we can determine that there are a range of time-series prediction models, each of which outperforms in distinct situations and has its own set of limitations. Although LSTM variants have been used, there have been limited reports of the Gated Recurrent Units (GRU) DL model. In addition, no DL model results have been mapped to a 2D choropleth map in order to visualise the effect of control measures. Besides, no application of reinforcement theory has been utilised for DL analysis. Furthermore, the DL models have not been linked directly to the Government website. To add to this, there is a lack of DL models that apply a combination of expert designed matrices and official statistics/government data to incorporate social demographic risk factors for modelling the effects of implementing various restriction measures.

In order to address the limitations of the existing system, the proposed work focuses on the analysis mapping of results from a reinforcement-based DL GRU model (trained with data including longitude and latitude coordinates) onto 2D choropleth maps in order to understand the effectiveness of various control measures. The proposed model is also linked to the Government UK website and an expert-curated matrix to incorporate effects of control measures and social demographic risk factors.[16] A web dashboard for the DL model was built. To the best of our knowledge, this is the first study to apply these techniques to include the examination of hotspot (high incidence) areas in the UK.



For peer review only - http://bmjopen.bmj.com/site/about/guidelines.xhtml

Here, the proposed work examines the 2D geographic trend based on simulations of various control measures at both the national and local authority (LA) levels in the UK in order to have a detailed understanding of the factors affecting the spread of COVID-19 at these levels as well as the potential impact of future policy measures. This knowledge would allow the UK government, LAs and individual citizens to make informed decisions about regional policies and personal exposure risks.

## METHODS

### Patient and public involvement

This research was done without patient and public involvement.

### Model Development

The proposed model enables predictions of the incidence and mortality related to COVID-19 in the upcoming five weeks and simulates the effect of control measures targeting the COVID-19 spread i.e. the number of facilities available for accommodation and food, pubs, retail shops, education, transport and storage, art, entertainment and recreational services, within each local authority region. The model also accounts for international migration inflow, internal migration inflow and outflow within the UK, thus simulating control measures that affect travel.

The proposed model is a Deep Recurrent Reinforced Learning (DRRL)-based model (supplementary material Part I) named National Coronavirus Global Forecast System (NCGFS) that combines the synergistic properties of GRU,[17] and



For peer review only - http://bmjopen.bmj.com/site/about/guidelines.xhtml

reinforcement deep learning.[18] Like other DL models, GRU has the ability to model non-linear and temporal relationships between and within high dimensions of variables. However, GRU is also expected to be well suited in small dataset scenarios and is computationally more efficient.[19] The reinforcement learning element of NCGFS enables it to adapt to newly inputted data and make more accurate forecasts.

All available LA data was split 80:20 into training and validation data subsets. Data were pre-processed using scaling - subtracting their corresponding mean and dividing by the standard deviation values. Following the completion of predictions, the prediction outputs are then scaled back to their original scale.

The NCGFS neural network model utilised an input layer, numerous hidden layers, and an output layer. A complex series of non-linear matrix computations are applied to the input data to relate the target output (i.e. cases and mortality) and to the other data columns (e.g. amount of international migration inflow or internal migration inflow and outflow within UK, number of retail shops etc.). The model is first trained using the existing data subsets provided through a data generator. Each column of the data is assigned a specific weight at each of the nodes in the hidden layers, and these weights are progressively updated to minimise the mean absolute error (MAE) between the predicted and actual values, using the RMSProp optimisation algorithm [20]. Selection for RMSProp is further detailed in Supplementary Material, Part I Recurrent Optimisation Algorithms. During the Prediction process, an input data matrix of the same dimension as the training data is then passed into the input layer. The neural network's hidden layers then use the weights learned during the training process to predict the most likely incidence and mortality based predictor variables from each corresponding week.



For peer review only - http://bmjopen.bmj.com/site/about/guidelines.xhtml

The final model consists of two components, model-M (master model) and model-R (reinforced model) that serve different purposes. Model-M accounts for the relationships of variables across different LAs, whilst model-R provides improved forecasting performance for each individual LA that are selected for analysis. For detailed specifications of model parameters, please see Supplementary Material, Part I Neural Network architecture and configuration. This model is particularly apt at generalisation and is capable of forecasting a wide range of LA simultaneously. The model uses model-R to increase forecast performance for the individual LA that are selected for analysis. Model-M is updated with several additional epochs of training data from the selected LA to reinforce and optimise the predictions. Software code is available through https://github.com/s0810110/Cvd_NCGFS_TrendAnalysis.

**Data Linkage**

The data used to train the deep learning model is based on various datasets that have the potential to influence the trend in the number of COVID-19 cases and mortality at the national and local level (refer to Supplementary Materials, Part II. **A.** for more details), including domains of deprivation,[21] number of bars and pubs,[22] business size,[23] population estimate (male, female, by age, overall),[24] etc.[25–27] We use the R language and the R SDK for COVID-19, i.e. a set of software commands to retrieve data remotely, as published by Public Health England, to automatically extract the latest daily cases and mortality figures for all LA within the UK. Using this approach, we are able to automate and dynamically predict the cases and mortality as new data is generated by GOV.UK.  We use R to convert these data from daily figures into weekly counts and link this data to the data described in Supplementary Materials, Part II. A.



For peer review only - http://bmjopen.bmj.com/site/about/guidelines.xhtml

Specifically, knowledge from experts in risk modelling is used to curate a matrix containing three indices that together are named the COVID-19 General Policy (CvdGPIc) indices: LockdownScore, QuarantineMeasures,[28] and SchoolOpening.[27,29] The main dataset is connected to these index scores based on the weeks each of the associated policies was implemented and the relative effects at each time period.

Furthermore, the number of tourists arriving in Spain from Jan 2020 to July 2020 were obtained and adjusted by the proportion of UK tourists in Spain from the year 2019. As data on international travel is not readily available for the period affected by COVID-19, the rationale is to use the amount of UK travel to Spain as an indicator for the impact of international travel on the spread of COVID-19,[30,31] since Spain is a frequent UK tourist destination. Our GRU model is not only trained on the above data but also includes the longitude and latitude of each local authority as part of the model.[32]

A secured web dashboard was developed that enables users to explore the adjustment effects of risk factors and control measures on the spread of COVID-19 and can be made available on request(http://137.222.198.54:8081/).

Whilst the LA boundaries data is not included in the training process, the main dataset is also linked to this data following forecast generation, so the deep learning model will also provide the prediction of the incidence or mortality in the next five weeks in a geographical map view. Furthermore, the model has the capability to toggle the map view by local authority or Public Health England regions. These views will not only be useful for the Government to see the future effects of different control measures changes but also for the individual citizens to understand their risk of movement within and between local regions in the upcoming future.



For peer review only - http://bmjopen.bmj.com/site/about/guidelines.xhtml

For analysis in the map view, the geographical regions from the top to bottom of England is divided into four equidistant slices, which we shall name slice n2, n1, s1, s2, respectively. These categories will be applied to all other geographical plots hereafter to facilitate discussion. The areas with a higher number of cases are shown in darker colours with 6 grades of severity (I – VI) covering the ranges 0-250 (I); 250-500 (II); 500-750 (III); 750-1000 (IV); 1000-1250 (V); 1250-1500 (VI). Any number outside of this range is shown in grey and is classed as grade VII.

**Model Validation**

The model is internally validated for the whole of England, whereby the model is trained using all data except for weeks 41 to 46. The data from this interval are evaluated using Root Mean Squared Error (RMSE), Mean Absolute Error (MAE), Mean Absolute Percentage Error (MAPE), and Correlation Coefficient.[11,12] Average ranking of performance metrics were performed as per Eqs. (24), (25).[11] At the time of this work, only data up to week (wk) 46 are available. The risk and control parameters are adjusted within the web dashboard from wk 40 onwards to enable predictions to simulate a full/national lockdown (FLD) from wk 45 onwards. This is because it is known that a FLD had been applied in the UK from wk 45 (00.01 on Thursday 5 November 2020), and prior to that, local lockdown with social distancing (LD_SD) had been implemented.[33]

During the revision of this work, data for week 51 had become available and was downloaded from GOV.UK to enable external validation of simulated results. This was performed for top 21 hotspots using LD_SD and FLD separately. The same statistic metrics were used as that for internal validation. The following section explains the model simulation process in more details.



For peer review only - http://bmjopen.bmj.com/site/about/guidelines.xhtml

## Model Simulation

Simulations are performed using the final model that is trained using the approach described in the Model Development section. All data i.e. from week 5 to 46 are included for training this model. The model is used to simulate the effects of numerous different COVID-19 prevention measures on the number of cases at week 51 i.e. 5 weeks ahead of the latest available data. The risk and control parameters that model the corresponding measures are set from week 40 onwards to enable predictions to simulate the implementation of various measures from week 45 onwards, rather than FLD, which was what the Government actually implemented. The measures simulated are: (a) No lockdown vs. local lockdown with social distancing (LD_SD); b) LD_SD vs. full/national lockdown (FLD); c) LD_SD vs. LD_SD with international travel -50%; d) LD_SD vs. LD_SD with closing school -50% e) LD_SD with travel quarantine 5.5 (see Supplementary Material, Part II. A., 11) vs. LD_SD with full travel quarantine 10; f) LD_SD with 100% pubs open vs. LD_SD with -50% pubs; (g) LD_SD with 100% food & accommodation services open vs. LD_SD with -50% food & accommodation services open; (h) LD_SD with -50% retail services open vs. LD_SD with 100% retail services open. For details on the implementation of these measures, please refer to Supplementary Materials, Part II. A.

These measures are simulated firstly for individual LA by selecting a baseline LA with a relatively low case count and comparing the effect of the measures when applied to a LA with a very high number of cases i.e. a hotspot area. The measures are then ranked by order of effectiveness. This is so that the relative effectiveness of each measure can be understood at the local level. Secondly, the measures are simulated for all the LA in England to visualise the relative effectiveness of each measure at a national level. For the 21 LA with the highest cases when using a



For peer review only - http://bmjopen.bmj.com/site/about/guidelines.xhtml

LD_SD measure, the predicted cases counts at week 51 are extracted and plotted to analyse the efficacy of each measure across these nationally “hard” to tackle areas. This comparison also enabled the ranking of the relative effectiveness of each measure at these hotspots.

# RESULTS

Model validation of predictions against actual results for week 46 showed a good match between the simulation and an actual number of cases across all the LA concerned (fig 1). The model distinguished the LA with high cases from the areas with a low number of cases (fig 2a, b). Furthermore, the model performs especially well for low-grade LA (Table 1). The tendency towards better performance in low degree LA, maybe because data from weeks 41 to 46 containing sharp changes in the trend have not been included. Good performance was achieved in terms of RMSE, MAE, MAPE, Correlation Coefficient and ranking when benchmarked against Devaraj et al.[11] and Melin et al.[12,12] (Supplementary Materials, Part III. Table S2., Table S3.). FLD simulation performed better than LS_SD in external validation using the top 21 hotspots. Results from simulation of cases and mortality up to week 51, using data from wks 41 to 46, are further discussed below.

The effects of different measures were first observed at a local level. Southampton was selected as a baseline for observing the effects of measure changes. As Southampton is a grade I LA with a low case number of 187 in week 46, the effects of measure changes were readily perceived with effectiveness ranked from most effective to least effective (Supplementary Materials, Part IV fig. S6): b) full lockdown; c) LD_SD & international travel -50%; e) LD_SD & 100% quarantine; d)



For peer review only - http://bmjopen.bmj.com/site/about/guidelines.xhtml

LD_SD & closing school -50%; f) LD_SD & closing pubs -50%. There were negligible differences observed between LD_SD and g) LD_SD & -50% food & Accommodation and h) LD_SD & -50% Retail.

As Leeds was in the highest grade (VII) for both week 46 (actual) and week 51 (predicted), it was selected for observing the effects of different measures on ‘hard’ to tackle areas. As the number of cases for Leeds as approximately 5 times higher than Southampton, the effect of measures relative to the number of cases in any week were much smaller in the former than the latter. For Leeds, no difference was observed for predicted cases at week 51 between no lockdown and LD_SD. Full lockdown (Supplementary Materials, Part IV. fig. S7b) was the most effective, followed by LD_SD with a reduction in international travel by 50%, although the effects were much less in proportion to the number of cases than Southampton. There was a negligible impact on the number of cases at week 51 for the remaining measures (fig. S7e-h).

Figure 2c shows the predicted cases in week 51 using LD_SD. At a national level, it can be seen that there would be a rapid rise in the number of cases, especially in the horizontal “belt” along the n1 region. In addition, there is at least one LA in each of the other slices n2, s1 and s2 that are expected to rise to grade VI or above. The majority of LA locations elsewhere, which were mostly at grade I in week 46, are expected to rise to grade II or III. The top 21 hotspots at week 51 using LD_SD were selected for subsequent analysis (Table 2).

LD_SD was shown (fig 3) to be effective in suppressing the increase in cases for Birmingham (-17%), Bradford (+0.98%), Kirklees (-6.6%) and Leicester (-1.3%). LD_SD was shown to be ineffective for suppressing the increase in cases for the remaining 17 LA, with the highest predicted rises for Wirral (325%), Stockport (163%), Tameside (188%), Rotherham (158%), Derby (130%).



For peer review only - http://bmjopen.bmj.com/site/about/guidelines.xhtml

**Table 1 Validation model: Number of actual and predicted cases and mortalities. The results show that there is a close match between the actual and predicted number of cases, especially for LA at grade III or below.**

| Local Authority | Number of Actual Cases for week 46 | Cases Forecast for week 46 | Number of Actual Mortalities for week 46 | Mortality Forecast for week 46 |
|---|---|---|---|---|
| Intervention | *Full Lockdown* | | | |
| Wolverhampton | 438 | 482 | 4 | 5 |
| Gedling | 179 | 196 | 6 | 2 |
| Welwyn Hatfield | 119 | 130 | 0 | 2 |
| Wiltshire | 201 | 219 | 1 | 4 |
| Portsmouth | 220 | 239 | 0 | 3 |
| Bromley | 217 | 232 | 2 | 3 |
| Stockton-on-Tees | 467 | 498 | 7 | 7 |
| Stockport | 517 | 550 | 13 | 8 |
| South Kesteven | 153 | 162 | 6 | 1 |
| Hammersmith and Fulham | 166 | 175 | 1 | 2 |
| Kingston upon Thames | 150 | 158 | 4 | 2 |
| Ribble Valley | 93 | 98 | 4 | 2 |
| East Cambridgeshire | 34 | 36 | 1 | 1 |
| Redcar and Cleveland | 380 | 396 | 7 | 4 |
| Sedgemoor | 55 | 57 | 3 | 1 |
| Cheshire East | 496 | 514 | 6 | 7 |
| Wealden | 76 | 79 | 1 | 2 |
| Charnwood | 371 | 382 | 3 | 3 |
| South Somerset | 72 | 74 | 1 | 1 |
| Southend-on-Sea | 137 | 140 | 0 | 3 |
| Chelmsford | 110 | 112 | 2 | 2 |
| Rushcliffe | 124 | 126 | 4 | 2 |
| Merton | 146 | 148 | 0 | 2 |
| Shropshire | 426 | 428 | 6 | 6 |
| Harrogate | 253 | 253 | 1 | 2 |
| Central Bedfordshire | 226 | 225 | 5 | 4 |
| Sutton | 155 | 154 | 5 | 3 |
| Oldham | 735 | 732 | 15 | 8 |
| Hillingdon | 325 | 323 | 3 | 3 |
| Basildon | 168 | 167 | 4 | 3 |
| Plymouth | 196 | 192 | 2 | 3 |
| Test Valley | 59 | 58 | 1 | 1 |
| Walsall | 605 | 590 | 15 | 6 |



For peer review only - http://bmjopen.bmj.com/site/about/guidelines.xhtml

For peer review only

| | | | | |
|---|---|---|---|---|
| Southampton | 187 | 182 | 0 | 2 |
| Selby | 129 | 124 | 2 | 1 |
| South Holland | 105 | 100 | 2 | 1 |
| Chiltern | 57 | 54 | 0 | 1 |
| Derbyshire Dales | 82 | 78 | 1 | 2 |
| Chichester | 58 | 54 | 0 | 1 |
| Barnet | 378 | 354 | 5 | 4 |
| Tameside | 447 | 417 | 18 | 12 |
| Salford | 577 | 537 | 17 | 7 |
| Havant | 77 | 71 | 1 | 1 |
| Waverley | 97 | 89 | 0 | 1 |
| Nuneaton and Bedworth | 247 | 226 | 4 | 3 |
| New Forest | 92 | 84 | 6 | 1 |
| Ryedale | 67 | 61 | 1 | 1 |
| Peterborough | 224 | 204 | 2 | 4 |
| North Hertfordshire | 89 | 81 | 1 | 1 |
| Epping Forest | 130 | 118 | 2 | 1 |

Note: only 50 LA are displayed. For validation data on all LA, please contact the authors.

## Table 2 Final model: number of actual and predicted cases and mortalities. Results are shown for the top 21 LA with the highest predicted cases observed at wk 51 using LD_SD.

| Local Authority | Number of Actual Cases for week 46 | Number of Actual Mortalities for week 46 | Cases Forecast for week 51 | Mortality Forecast for week 51 | Cases Forecast for week 51 | Mortality Forecast for week 51 |
|---|---|---|---|---|---|---|
| Intervention | *Full Lockdown* | | *Local lockdown with social distancing* | | *Full lockdown* | |
| Leeds | 1801 | 17 | 1881 | 21 | 499 | 17 |
| Sheffield | 948 | 36 | 1784 | 32 | 275 | 14 |
| Birmingham | 1957 | 35 | 1627 | 25 | 537 | 17 |
| Wigan | 759 | 34 | 1554 | 24 | 346 | 10 |
| Manchester | 1067 | 13 | 1550 | 20 | 427 | 13 |
| Bradford | 1534 | 24 | 1549 | 20 | 722 | 17 |
| Stockport | 517 | 13 | 1529 | 17 | 218 | 7 |
| Liverpool | 750 | 29 | 1509 | 22 | 234 | 8 |
| Rotherham | 561 | 20 | 1448 | 22 | 257 | 7 |
| Kingston upon Hull | 1011 | 21 | 1368 | 18 | 584 | 12 |
| Oldham | 735 | 15 | 1336 | 17 | 509 | 11 |
| Wirral | 311 | 13 | 1324 | 19 | 65 | 4 |
| Bolton | 635 | 18 | 1299 | 17 | 447 | 11 |
| Bristol | 763 | 8 | 1296 | 14 | 444 | 13 |
| Tameside | 447 | 18 | 1288 | 20 | 255 | 7 |



For peer review only - http://bmjopen.bmj.com/site/about/guidelines.xhtml

For peer review only

| | | | | | | |
|---|---|---|---|---|---|---|
| County Durham | 1161 | 23 | 1252 | 26 | 377 | 7 |
| Derby | 537 | 11 | 1234 | 15 | 335 | 8 |
| Walsall | 605 | 15 | 1233 | 21 | 288 | 7 |
| Kirklees | 1292 | 25 | 1207 | 29 | 557 | 14 |
| Leicester | 1006 | 7 | 993 | 15 | 581 | 11 |
| Sandwell | 762 | 21 | 969 | 25 | 398 | 10 |

LD_SD with -50% international travel was the most effective measure after full lockdown (blue vs brown, fig 4). 100% quarantine (pink) was the next most effective supplementary measure, with similar effectiveness to international travel -50% except for three LA. Notably, LD_SD with 100% quarantine resulted in higher cases than LD_SD with international travel -50% for Bradford (+9.1%), Leicester (+7.6%). As an exception, Manchester had -41% fewer cases when using the quarantine measure compared to international travel restrictions.

The supplementary effect of school closing -50% was less than international travel restrictions for all 21 LA, with the number of cases being (+9.2%) higher on average using the former measure. Closing pubs -50% had a similar, albeit slightly lower level of effectiveness compared to school closing, with a higher number of cases (+2.2%) on average using the former measure compared to the latter. Again, reducing the number of food & accommodation services -50% had a similar, but a slightly lower level of effectiveness compared to pubs closing, with the number of cases (+2.0%) being higher on average using the former measure. In addition, a reduction in the number of retail services -50% resulted in a similar effect to food & accommodation services -50%, with on average a minimal increase in the number of cases (+0.29%) using the former measure. It can be seen that, on average, the ranking of measure effectiveness for the national hotspots are the same as the local baseline, i.e. Southampton.



For peer review only - http://bmjopen.bmj.com/site/about/guidelines.xhtml

# DISCUSSION

Previous studies have evaluated the prediction performances of DL and non-DL based models without 2D choropleth analysis and found that SLSTM outperformed other models because of better hyperparameter tuning and reduction in bias. In addition, it was found that ARIMA outperforms LSTM model.[11] Using the same average ranking metrics, we found that NCGFS (Overall Rank 1-3) outperformed SLSTM (Overall Rank 4), ARIMA (Overall Rank 5), and LSTM (Overall Rank 6). However, there are several limitations of this comparison 1) predictions are for different countries; 2) lower mortality rates in England compared to India may bias better ranking towards NCGFS; 3) comparison does not account for recovered cases. NCGFS also demonstrated good performance in terms of case prediction when benchmarked against MNNF in terms of RMSE (700.88 vs 1554.03).[12] Whilst, a few studies have analysed 2D geospatial predictions, e.g. by converting to heatmaps,[34] and considered hotpot regions, these have mainly been using either modified regression or differential equation techniques rather than DL-based techniques.[10,35,36] Although interactive dashboards have been developed for tracking COVID-19,[37] these typically do not enable prediction through simulation of control measures. Furthermore, although Reinforcement Learning has been applied, it has not typically been combined with 2D map analysis or lack external validation.[38,39] In this article, we demonstrate the use of a reinforcement-based DL GRU model with 2D choropleth maps to analyse spatial representation of results in order to rank the efficacy of various control measures. The proposed model is embedded in an interactive dashboard linked to the Government UK website and an expert-curated matrix to incorporate effects of control measures and social demographic risk factors. This combination of techniques has not yet been



For peer review only - http://bmjopen.bmj.com/site/about/guidelines.xhtml

widely used, and it provides a number of advantages over each individual formulation alone. The NCGFS model can be used to make inferences into the effectiveness of different measures at both the national and local levels. The model suggests that there is variation in the effect of each measure across different regions. Notably, our results indicate that the protective effects of lockdown measures benefit some local authorities more than others (Supplementary Material, Part IV. fig S6 and S7) and that local lockdown with social distancing is ineffective compared to national lockdown in suppressing the increase in cases for most of the local authority areas. That is, if the government had kept the same local lockdown with social distancing policies, which they had implemented from week 40 onwards rather than switching to a national lockdown policy at week 45, then we would have seen a rapid rise in cases not only in the n1 belt region, but also in areas such as County Durham (n2), Bristol (s2) and Birmingham (s1), as well as in many other areas across England.

Local lockdown with social distancing and without additional measures may be inefficient in stopping rapid rise of hotpot regions due to the geographical properties of hotspot regions. Hotspots along the middle of the n1 geographical slice constitute a tight cluster of large metropolitan cities. The high number of cases may be partly attributed to the high number of services such as pubs and schools available and the amount of travel in these areas. We expect that this effect could possibly be enhanced by the fact that the n1 slice contains a large number of LA areas with many boundary links to other hotspots, which agrees with another study that highlighted influences of neighbouring small areas and found continuous bands of hotspot regions.[36]

Since the government is only able to impose a national lockdown for a limited period, follow-up measures should incorporate LD_SD with additional measures as LD_SD  alone is likely not to be sufficient. The actual FLD had lasted only up to Dec



For peer review only - http://bmjopen.bmj.com/site/about/guidelines.xhtml

2 2020 (wk 49), after which LD_SD was implemented by the government. The UK Government had implemented LD_SD for two weeks in between wk 49 and wk 51. As a substantially larger proportion of weeks between wk 45 and wk 51 were implemented using FLD rather than LD_SD, we found the FLD simulation (Overall Rank 2) better predicted actual results than LD_SD (Overall Rank 3) as expected. Trend and 2D map analysis showed that introduction of additional measures on top of local lockdown with social distancing can help to suppress the increase of or even decrease the number of cases in national hotspots as well as local areas where cases are not very high. Our model shows that the ranking of the average effectiveness of each supplementary measure is consistent across the national hotspots and local baseline, and this ranking can be used to prioritise those interventions according to an order of effectiveness. Nonetheless, it was also observed that specific measures are more effective for some LA compared to others. In these cases, it is necessary to adjust the priorities of the measures implemented accordingly.

The model has highlighted the importance of reducing the amount of international travel, the number of open schools and pubs as well as the implementation of travel quarantine procedures in controlling the spread of COVID-19 over other measures, such as reducing the number of food & accommodation and retail services, which seemed less relevant on the virus spread (fig 4 and fig S6). Our finding of the usefulness of restricting international travel and applying travel quarantine is in contrast with another study, which found that quarantine of travel from endemic countries was not effective.[4] One explanation for international travel being more impactful than travel quarantine is that whilst travel quarantine provide the government with some control over which countries to enforce a 14-day self-isolation



For peer review only - http://bmjopen.bmj.com/site/about/guidelines.xhtml

on travellers' return, it provides little control over the activities of travelling individuals once they arrive at their destinations as well as the level of preventative health measures at those destinations. Furthermore, the individuals who travel are more likely to encounter places of gathering whilst abroad. In addition, even with COVID-19 testing in place, the journey from the airport back to the home of the traveller allows an increased opportunity of spreading the virus, particularly if public transport such as taxis or buses are used.[4,8] Therefore, these measures are not as direct as limiting the amount of international travel.

Whilst closing schools were not as effective as international travel and quarantine restrictions, we found this measure to be more effective than closing pubs. One potential explanation for this is that schools are more crowded places and are subject to a more frequent number of close contact scenarios in comparison to the pub. The view that schools contribute to the spread of COVID-19 has been supported by the literature.[40,41] Whilst the virus may pose a low risk of mortality to the children themselves, these frequently asymptomatic carriers can also lead to the spread of the virus to their households, teachers and communities.

The reason why minimal effects were found for food & accommodation and retail restrictions may be because in these sectors, people generally associate with others that they are closely associated with. For example, families are more likely to sit with each other in restaurants or walk together when shopping rather than with people they are less familiar with. This is not the case in pubs as anyone from the communal area can be present.

It is unexpected that in the s2 slice that Bristol has higher predicted cases than the LA in the London area as one would have thought the latter comprising a total population of 9 million (2019) and a high traffic volume owing to its large underground



For peer review only - http://bmjopen.bmj.com/site/about/guidelines.xhtml

network system would result in much higher case numbers. We expect that this may be because the London region LA generally has less health and disability deprivation (Deciles: Wandworth: 7; Barnet: 8.9; Brent: 7.3; Waltham Forest: 6.1) compared to Bristol (Decile: 4.4). This is supported by the findings which suggest that existing comorbidities are associated with an increased likelihood of COVID-19 hospital admission.[42]

In light of evidence given by the comparison between the LA within the London region and Bristol, we expect the effect of LA boundary connections to be adjusted by the degree of health and disability deprivation. Indeed, we found that regions with a high number of cases along the horizontal "belt" in the n1 region had a high degree of health and disability deprivation (Decile: Manchester: 1.9; Leeds: 4.1; Bradford: 3.3; Liverpool: 1.8; Sheffield: 3.9; Wigan: 3.4). Another study reports a similar result.[36] This also applies to County Durham (n2), which has a high degree of health and disability deprivation (decile: 2.9) and was seen to have a significant increase in cases at week 51 using a LD_SD measure.

One limitation of our study is that for simulated week 51, there was an LA in Kent that was coloured grey on the map but were in fact, reporting negative values. A future improvement would be to transform or limit the prediction outputs to retain meaningful information whilst preventing negative values. Nonetheless, the result is interesting and giving the LA a grade of VII may still be valid, since the highly infectious Kent variant emerged in week 39 (20 September).[43] The present study has not specifically dealt with the modelling of variants. In addition, vaccination effects could not be accounted for as these were only beginning to be rolled out (Dec 2, wk 49) at the point of this study.[44] Whilst a variety of data sources have been used, this study has not analysed the effect of in-hospital admission or clinical comorbidity. These are



For peer review only - http://bmjopen.bmj.com/site/about/guidelines.xhtml

issues that deserve to be further explored. Future work could be done to compare the current model against those found in similar studies e.g. ARIMA, LSTM, Stacked LSTM (SLSTM), Prophet,[11], Bayesian hierarchical space–time SEIR model,[36] NAR and FITNET neural networks, using the same dataset.[12] Furthermore, a Hybrid Q-learning based algorithm could be used whereby these models represent potential actions to update the Q cumulative reward matrix.[39] Whilst the current model takes a risk factor matrix as one of its inputs and this was built with expert input, a fuzzy logic approach with functionally modelled inputs and outputs was not utilised.[15] Incorporating such methods could enhance the interpretability of the risk factor matrix.

# CONCLUSION

This study highlights the importance of simulating the effects of various control measures using map and non-map-based analyses to prioritise COVID-19 preventative measures. This was demonstrated for both local hotspot zones and on a nationwide scale. Furthermore, at the LA level we demonstrated the utility of geographical slicing for comparative analysis of interventional effects across time periods, and thereby can also allow governments to assess the optimal measures to apply. It is advisable to assess the effectiveness of lockdown with social distancing alone against that when combined with other adjunctive measures and implement periodic monitoring at both trend and map dimensions to reduce the risk of outbreak rebound following lockdown easing. Lastly, this study highlights the importance of controlling international travel, and this should be further explored with the comparative analysis of effectiveness against newly developed vaccine measures.



For peer review only - http://bmjopen.bmj.com/site/about/guidelines.xhtml

# FOOTNOTES

Contributors: TD and UB conceived and designed this study. TD and UB acquired the data. TD, UB, SS, DF and AD analysed and interpreted the data. MC, DF, JC and GDA provided administrative and operational support. The initial manuscript was drafted by TD; all authors critically revised the manuscript and approved its final version. DF made a substantial contribution to the revision of the manuscript. UB acts as guarantor. The corresponding author attests that all listed authors meet authorship criteria and that no others meeting the criteria have been omitted.

---

Copyright/license: *I, the Submitting Author has the right to grant and does grant on behalf of all authors of the Work (as defined in the below author licence), an exclusive licence and/or a non-exclusive licence for contributions from authors who are: i) UK Crown employees; ii) where BMJ has agreed a CC-BY licence shall apply, and/or iii) in accordance with the terms applicable for US Federal Government officers or employees acting as part of their official duties; on a worldwide, perpetual, irrevocable, royalty-free basis to BMJ Publishing Group Ltd ("BMJ") its licensees and where the relevant Journal is co-owned by BMJ to the co-owners of the Journal, to publish the Work in BMJ Open and any other BMJ products and to exploit all rights, as set out in our licence.*

*The Submitting Author accepts and understands that any supply made under these terms is made by BMJ to the Submitting Author unless you are acting as an*



For peer review only - http://bmjopen.bmj.com/site/about/guidelines.xhtml

*employee on behalf of your employer or a postgraduate student of an affiliated institution which is paying any applicable article publishing charge ("APC") for Open Access articles. Where the Submitting Author wishes to make the Work available on an Open Access basis (and intends to pay the relevant APC), the terms of reuse of such Open Access shall be governed by a Creative Commons licence – details of these licences and which Creative Commons licence will apply to this Work are set out in our licence referred to above.*

---

Funding: This study was supported by the NIHR Biomedical Research Centre at University Hospitals Bristol and Weston NHS Foundation Trust and the University of Bristol and the British Heart Foundation.. The funders had no role in the study design, data collection and analysis, decision to publish, or preparation of the manuscript. All authors had full access to all of the data (including statistical reports and tables) in the study and can take responsibility for the integrity of the data and the accuracy of the data analysis.

---

Competing interests: All authors have completed the Unified Competing Interest form (available on request from the corresponding author) and declare: no support from any organisation for the submitted Work; no financial relationships with any organisations that might have an interest in the submitted Work in the previous three years; no other relationships or activities that could appear to have influenced the submitted Work.



For peer review only - http://bmjopen.bmj.com/site/about/guidelines.xhtml

Ethical approval: Not needed

Transparency: The manuscript's guarantor affirms that the manuscript is an honest, accurate, and transparent account of the study being reported; that no important aspects of the study have been omitted; and that any discrepancies from the study as planned (and, if relevant, registered) have been explained.

Data sharing: All the data used in the study are available from public resources. The dataset is found in the Supplementary Materials document and can also be made available on request from the corresponding author.

## REFERENCES

1 COVID-19 cases worldwide by day. Statista. https://www.statista.com/statistics/1103040/cumulative-coronavirus-covid19-cases-number-worldwide-by-day/ (accessed 11 Dec 2020).

2 Dyer O. Covid-19: Denmark to kill 17 million minks over mutation that could undermine vaccine effort. *BMJ* 2020;**371**. doi:10.1136/bmj.m4338

3 Elavarasan RM, Pugazhendhi R, Shafiullah GM, *et al.* A hover view over effectual approaches on pandemic management for sustainable cities – The endowment of



For peer review only - http://bmjopen.bmj.com/site/about/guidelines.xhtml

prospective technologies with revitalization strategies. *Sustain Cities Soc* 2021;**68**:102789. doi:10.1016/j.scs.2021.102789

4 Kumaravel SK, Subramani RK, Jayaraj Sivakumar TK, *et al.* Investigation on the impacts of COVID-19 quarantine on society and environment: Preventive measures and supportive technologies. *3 Biotech* 2020;**10**:393. doi:10.1007/s13205-020-02382-3

5 Varotsos CA, Krapivin VF. A new model for the spread of COVID-19 and the improvement of safety. *Saf Sci* 2020;**132**:104962. doi:10.1016/j.ssci.2020.104962

6 Sun T, Wang Y. Modeling COVID-19 epidemic in Heilongjiang province, China. *Chaos Solitons Fractals* 2020;**138**:109949. doi:10.1016/j.chaos.2020.109949

7 Varotsos CA, Krapivin VF, Xue Y. Diagnostic model for the society safety under COVID-19 pandemic conditions. *Saf Sci* 2021;**136**:105164. doi:10.1016/j.ssci.2021.105164

8 Madurai Elavarasan R, Pugazhendhi R. Restructured society and environment: A review on potential technological strategies to control the COVID-19 pandemic. *Sci Total Environ* 2020;**725**:138858. doi:10.1016/j.scitotenv.2020.138858

9 Madurai Elavarasan R, Shafiullah G, Raju K, *et al.* COVID-19: Impact analysis and recommendations for power sector operation. *Appl Energy* 2020;**279**:115739. doi:10.1016/j.apenergy.2020.115739

10 Melin P, Monica JC, Sanchez D, *et al.* Analysis of Spatial Spread Relationships of Coronavirus (COVID-19) Pandemic in the World using Self Organizing Maps. *Chaos Solitons Fractals* 2020;**138**:109917. doi:10.1016/j.chaos.2020.109917

11 Devaraj J, Madurai Elavarasan R, Pugazhendhi R, *et al.* Forecasting of COVID-19 cases using deep learning models: Is it reliable and practically significant? *Results Phys* 2021;**21**:103817. doi:10.1016/j.rinp.2021.103817

12 Melin P, Monica JC, Sanchez D, *et al.* Multiple Ensemble Neural Network Models with Fuzzy Response Aggregation for Predicting COVID-19 Time Series: The Case of Mexico. *Healthcare* 2020;**8**:181. doi:10.3390/healthcare8020181

13 Castillo O, Melin P. Forecasting of COVID-19 time series for countries in the world based on a hybrid approach combining the fractal dimension and fuzzy logic. *Chaos Solitons Fractals* 2020;**140**:110242. doi:10.1016/j.chaos.2020.110242

14 Castillo O, Melin P. A Novel Method for a COVID-19 Classification of Countries Based on an Intelligent Fuzzy Fractal Approach. *Healthcare* 2021;**9**:196. doi:10.3390/healthcare9020196

15 Castillo O, Melin P. A new fuzzy fractal control approach of non-linear dynamic systems: The case of controlling the COVID-19 pandemics. *Chaos Solitons Fractals* 2021;**151**:111250. doi:10.1016/j.chaos.2021.111250



For peer review only - http://bmjopen.bmj.com/site/about/guidelines.xhtml

16 Benedetto U, Dimagli A, Gibbison B, *et al.* Disparity in clinical outcomes after cardiac surgery between private and public (NHS) payers in England. *Lancet Reg Health – Eur* 2021;**1**. doi:10.1016/j.lanepe.2020.100003

17 Cho K, van Merrienboer B, Bahdanau D, *et al.* On the Properties of Neural Machine Translation: Encoder-Decoder Approaches. *ArXiv14091259 Cs Stat* Published Online First: 7 October 2014.http://arxiv.org/abs/1409.1259 (accessed 10 Dec 2020).

18 Mnih V, Kavukcuoglu K, Silver D, *et al.* Human-level control through deep reinforcement learning. *Nature* 2015;**518**:529–33. doi:10.1038/nature14236

19 Chung J, Gulcehre C, Cho K, *et al.* Empirical Evaluation of Gated Recurrent Neural Networks on Sequence Modeling. *ArXiv14123555 Cs* Published Online First: 11 December 2014.http://arxiv.org/abs/1412.3555 (accessed 11 Nov 2021).

20 Zou F, Shen L, Jie Z, *et al.* A Sufficient Condition for Convergences of Adam and RMSProp. In: *2019 IEEE/CVF Conference on Computer Vision and Pattern Recognition (CVPR)*. Long Beach, CA, USA: : IEEE 2019. 11119–27. doi:10.1109/CVPR.2019.01138

[dataset] 21 English indices of deprivation 2019. GOV.UK. https://www.gov.uk/government/statistics/english-indices-of-deprivation-2019 (accessed 2 Dec 2021).

[dataset] 22 Public houses and bars by local authority - Office for National Statistics. https://www.ons.gov.uk/businessindustryandtrade/business/activitysizeandlocation/datasets/publichousesandbarsbylocalauthority (accessed 2 Dec 2021).

[dataset] 23 UK business: activity, size and location - Office for National Statistics. https://www.ons.gov.uk/businessindustryandtrade/business/activitysizeandlocation/datasets/ukbusinessactivitysizeandlocation (accessed 2 Dec 2021).

[dataset] 24 Estimates of the population for the UK, England and Wales, Scotland and Northern Ireland - Office for National Statistics. https://www.ons.gov.uk/peoplepopulationandcommunity/populationandmigration/populationestimates/datasets/populationestimatesforukenglandandwalesscotlandandnorthernireland (accessed 2 Dec 2021).

[dataset] 25 Local Authority Districts (December 2019) Boundaries UK BUC. https://geoportal.statistics.gov.uk/datasets/local-authority-districts-december-2019-boundaries-uk-buc (accessed 2 Dec 2021).

[dataset] 26 Local Authority District to Public Health England Centre to Public Health England Region (December 2019) Lookup in England. https://geoportal.statistics.gov.uk/datasets/local-authority-district-to-public-health-england-centre-to-public-health-england-region-december-2019-lookup-in-england (accessed 2 Dec 2021).



For peer review only - http://bmjopen.bmj.com/site/about/guidelines.xhtml

[dataset] 27 Actions for schools during the coronavirus outbreak. GOV.UK. https://www.gov.uk/government/publications/actions-for-schools-during-the-coronavirus-outbreak (accessed 2 Dec 2021).

[dataset] 28 The UK's coronavirus timeline. Week UK. https://www.theweek.co.uk/107044/uk-coronavirus-timeline (accessed 2 Dec 2021).

[dataset] 29 Coronavirus: UK schools, colleges and nurseries to close from Friday. BBC News. 2020.https://www.bbc.com/news/uk-51952314 (accessed 2 Dec 2021).

[dataset] 30 Monthly tourist arrivals in Spain 2020. Statista. https://www.statista.com/statistics/1130775/number-of-monthly-arrivals-short-stay-accommodation-in-spain/ (accessed 2 Dec 2021).

[dataset] 31 Spain: number of tourists by country. Statista. https://www.statista.com/statistics/447683/foreign-tourists-visiting-spain-by-country-of-residence/ (accessed 2 Dec 2021).

[dataset] 32 Public Health England Centres (December 2016) Ultra Generalised Clipped Boundaries in England. https://geoportal.statistics.gov.uk/datasets/public-health-england-centres-december-2016-ultra-generalised-clipped-boundaries-in-england (accessed 2 Dec 2021).

33 Timeline of UK government coronavirus lockdowns. Inst. Gov. 2021.https://www.instituteforgovernment.org.uk/charts/uk-government-coronavirus-lockdowns (accessed 23 Nov 2021).

34 Khan S, Alarabi L, Basalamah S. Toward Smart Lockdown: A Novel Approach for COVID-19 Hotspots Prediction Using a Deep Hybrid Neural Network. *Computers* 2020;**9**:99. doi:10.3390/computers9040099

35 Islam A, Sayeed MdA, Rahman MdK, *et al.* Geospatial dynamics of COVID-19 clusters and hotspots in Bangladesh. *Transbound Emerg Dis* 2021;**68**:3643–57. doi:10.1111/tbed.13973

36 Sartorius B, Lawson AB, Pullan RL. Modelling and predicting the spatio-temporal spread of COVID-19, associated deaths and impact of key risk factors in England. *Sci Rep* 2021;**11**:5378. doi:10.1038/s41598-021-83780-2

37 Dong E, Du H, Gardner L. An interactive web-based dashboard to track COVID-19 in real time. *Lancet Infect Dis* 2020;**20**:533–4. doi:10.1016/S1473-3099(20)30120-1

38 Kumar RL, Khan F, Din S, *et al.* Recurrent Neural Network and Reinforcement Learning Model for COVID-19 Prediction. *Front Public Health* 2021;**9**:1437. doi:10.3389/fpubh.2021.744100

39 Khalilpourazari S, Hashemi Doulabi H. Designing a hybrid reinforcement learning based algorithm with application in prediction of the COVID-19 pandemic



For peer review only - http://bmjopen.bmj.com/site/about/guidelines.xhtml

in Quebec. *Ann Oper Res* Published Online First: 3 January 2021. doi:10.1007/s10479-020-03871-7

40 Sheikh A, Sheikh A, Sheikh Z, *et al.* Reopening schools after the COVID-19 lockdown. *J Glob Health*;**10**. doi:10.7189/jogh.10.010376

41 Stein-Zamir C, Abramson N, Shoob H, *et al.* A large COVID-19 outbreak in a high school 10 days after schools' reopening, Israel, May 2020. *Eurosurveillance* 2020;**25**:2001352. doi:10.2807/1560-7917.ES.2020.25.29.2001352

42 Price-Haywood EG, Burton J, Fort D, *et al.* Hospitalization and Mortality among Black Patients and White Patients with Covid-19. *N Engl J Med* 2020;**382**:2534–43. doi:10.1056/NEJMsa2011686

43 Mahase E. Covid-19: What have we learnt about the new variant in the UK? *BMJ* 2020;**371**:m4944. doi:10.1136/bmj.m4944

44 Coronavirus vaccine rollout. Inst. Gov. 2021.https://www.instituteforgovernment.org.uk/explainers/coronavirus-vaccine-rollout (accessed 26 Nov 2021).

# LEGENDS

Fig 1 Validation of cases for week 46 with weeks 41 to 46 excluded from data

Fig 2 Geographical level of cases for actual and predicted results based on different measures. a) exemplifies the use of geographical slices n2, n1, s1, s2. Additional results are available in Supplementary Materials, Part II. B.

Fig 3 For the top 21 LA with the highest predicted cases observed at wk 51 using LD_SD, plots were generated to compare the effects of full lockdown against LD_SD in terms of cases a) and mortalities b).

For peer review only

Fig 4 For the top 21 LA with the highest predicted cases observed at wk 51 using LD_SD, a plot is generated to compare the effect on the number of cases using a combination of LD_SD with other “supplementary“ measures.

For peer review only



For peer review only - http://bmjopen.bmj.com/site/about/guidelines.xhtml

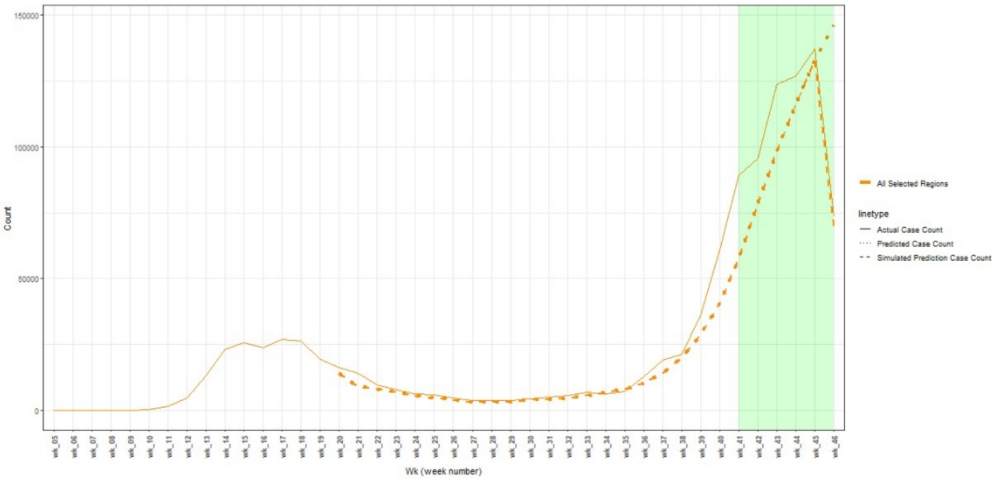


Fig 1 Validation of cases for week 46 with weeks 41 to 46 excluded from data

141x68mm (300 x 300 DPI)

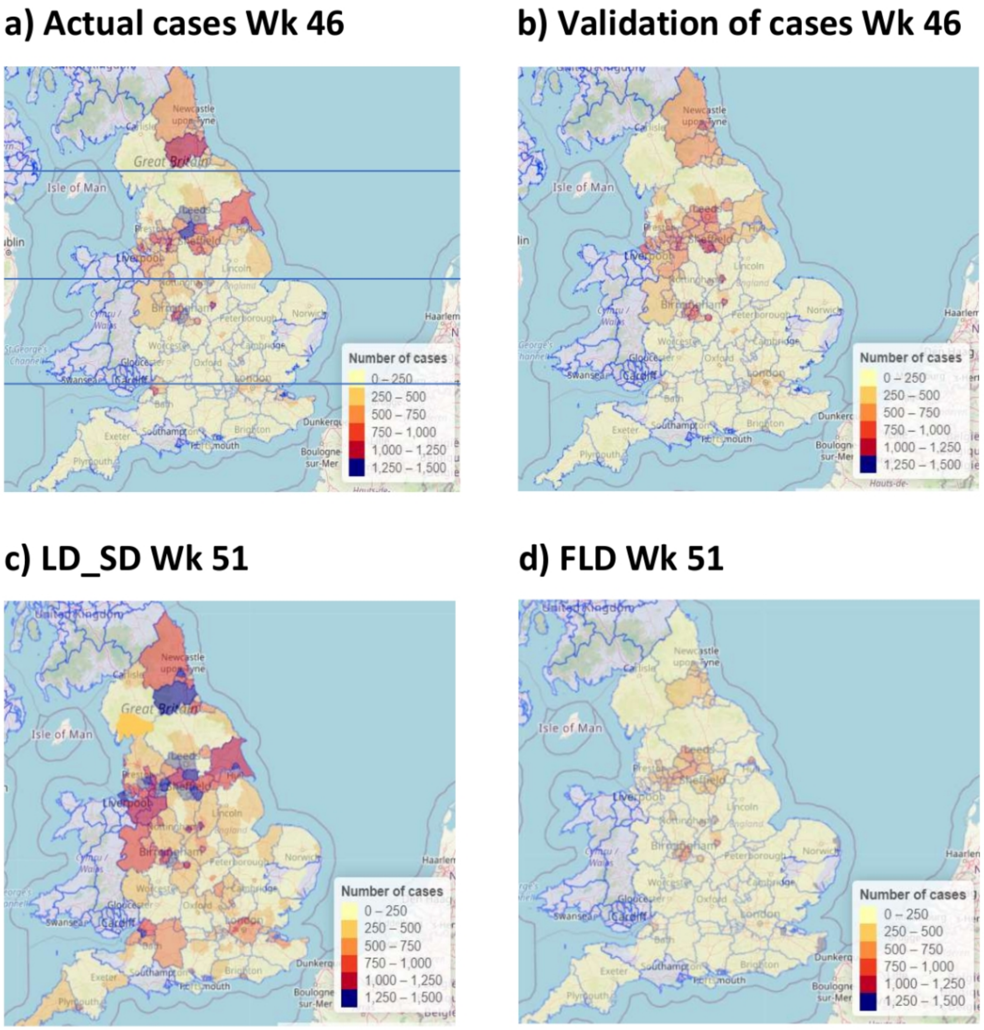


Fig 2 Geographical level of cases for actual and predicted results based on different measures. a) exemplifies the use of geographical slices n2, n1, s1, s2. Additional results are available in Supplementary Materials, Part II. B.

102x106mm (300 x 300 DPI)

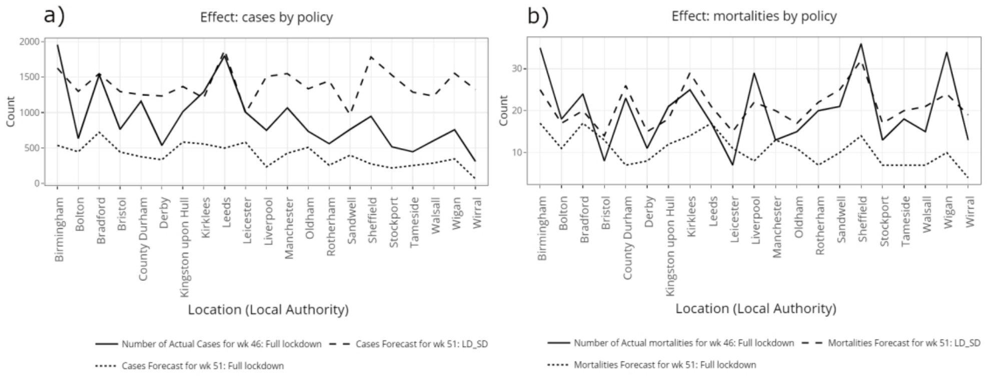


Fig 3 For the top 21 LA with the highest predicted cases observed at wk 51 using LD_SD, plots were generated to compare the effects of full lockdown against LD_SD in terms of cases a) and mortalities b).

151x57mm (300 x 300 DPI)

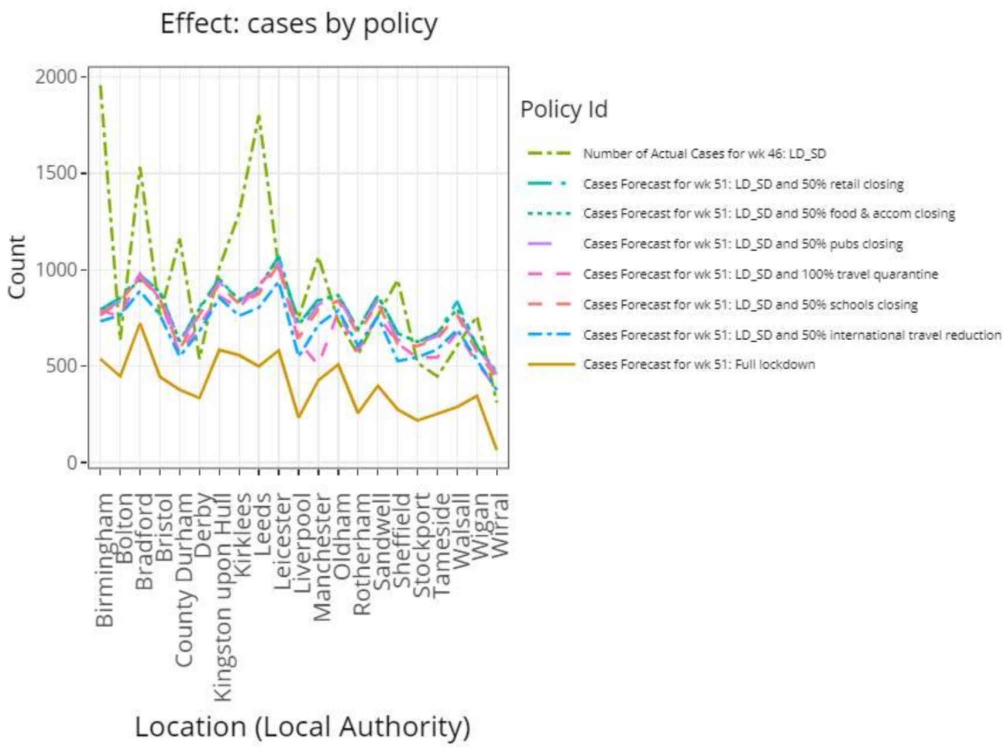


Fig 4 For the top 21 LA with the highest predicted cases observed at wk 51 using LD_SD, a plot is generated to compare the effect on the number of cases using a combination of LD_SD with other "supplementary" measures.

155x115mm (300 x 300 DPI)

# Supplementary Materials: A Deep Recurrent Reinforced Learning model to compare the efficacy of targeted local vs national measures on the spread of COVID-19 in the UK

*Tim Dong (0000-0003-1953-0063), Umberto Benedetto (0000-0002-7074-7949), Shubhra Sinha, Daniel Fudulu, Arnaldo Dimagli, Jeremy Chan, Massimo Caputo, Gianni Angelini*

Part I.

## GRU Gated Recurrent Units Model

We have chosen a Recurrent Neuro Network (RNN) category of model because of the ability of this type of model to model not only non-linear relationships between high dimension of variables, but also because of its ability to model temporal relationships within any of the variables considered. As figure S1. shows, the RNN model consists of cells is analogous to each unit of the traditional neuro network. Whilst traditional neuro network models accumulate weights $W_i$ (input to hidden layer) and $W_h$ (hidden to hidden layer), they do not calculate any recurrent weights $W_r$ that represent temporal relationships of each variable in the dataset. This recurrent type of weight is present in RNN models and hence is desirable for modelling a time dependent COVID-19 dataset.

For peer review only

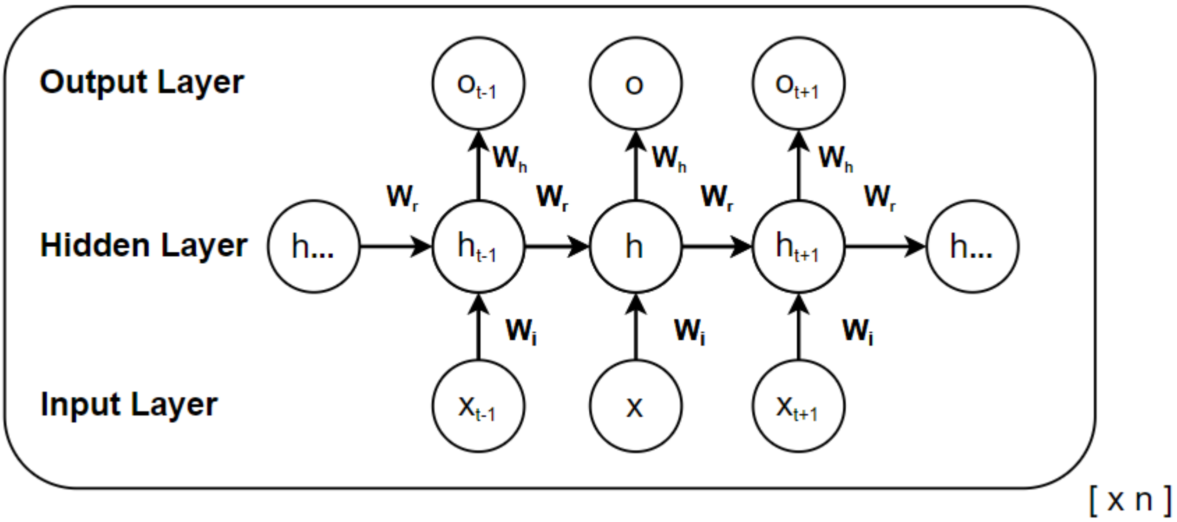


Figure S1. An RNN cell

GRU is a specific type of Recurrent Neuro Network (RNN) that incorporates long term memory and effectively deals with the vanishing gradient and gradient explosion problems that have affected various other categories of RNN. The GRU model was also selected because of its ease to optimise and its low computational cost in comparison to the LSTM model.

Unlike the LSTM model, which uses four gates, the GRU model uses only a single decision gate that controls both the update and reset of weights. The update gate is similar to an OR gate in electronics that either retains the state at the previous step $h_{t-1}$ or updates the previous state based on variable values $X_t$ provided at the current step. The reset gate is much like a resistor in that it controls that size of the effect from previous step that contributes to the current step.

For peer review only

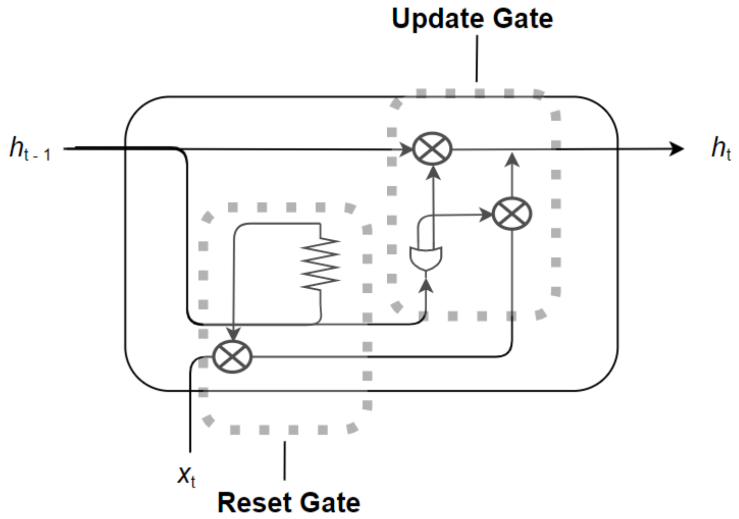


Figure S2. A GRU cell

The above diagram above provides an intuitive understanding into the processes that occur within a cell or node of a GRU model. The specific mathematical equation of the GRU model is given below:

$$h_t^i = z_t^i . h_{t-1}^i + \left(1 - z_t^i\right) tanh\left| b^i + \sum_j W_I^{i,j} x_t^j + \sum_j W_R^{i,j} r_{t-1}^j h_{t-1}^j \right|$$
$$z_t^i = \sigma\left( b_z^i + \sum_j W_z^{i,j} x_t^j + \sum_j W_z^{i,j} h_{t-1}^j \right)$$
$$r_t^j = \sigma\left( b_r^i + \sum_j W_r^{i,j} x_t^j + \sum_j W_r^{i,j} h_{t-1}^j \right)$$

where z represents for the update gate and r represents the reset gate, $W_I$ are the weights from the input to hidden layer, $W_R$ are the weights from the recurrent weights, b is the bias, $\sigma$ and tanh are the sigmoid and tanh activation functions respectively, $X_t$ are the inputs at time t and $h_{t-1}$ was the input from previous time step.

**Deep Reinforcement Learning**

Deep reinforcement learning is the application of reinforcement learning to neuro networks. The application of this approach has been exemplified in the field of computer gaming.[1] Essentially, the approach involves an agent that represents the computer system, which performs an action in the environment that it interacts with.

The environment is changed following the action and a reward is provided to the agent such that the action it selects from a list of actions or policy $\pi$ is optimised in any subsequent interactions with the environment.

Here, we present a technique whereby the NCGFS model represents the agent. It is initially presented with a set of observations $O_t$ from the national environment, $s$. Each individual observation has the property o ∈ $A$, where $A$ is the set of all the LA. Rather than using the traditional reward variable $r$, we use $L_t$ to represent the loss at the initial training phase $t$ of the deep learning model, i.e. at the generation point of model-M. Subsequently, when the agent is presented with the input observations $O_{t+1}$ that belongs to a single local authority, $s_{t+1}$ from the set A, it uses the action or in this case forecast from the list of potential forecasts that previously minimised the $L_t$ for $O_t$ to make the forecast $f_t$ for $O_{t+1}$. This induces a loss $L_{t+1}$ that is used to update the NCGFS model's policy decisions for the actual prediction of $O_{t+1}$ as $f_{t+1}$. The resulting model is termed model-R.

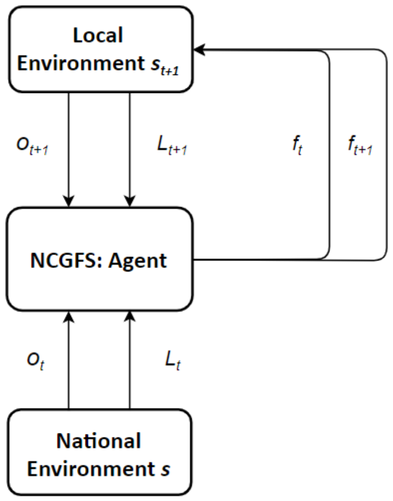


Figure S3. illustration of the Deep Reinforcement Learning aspect of NCGFS at the local authority level.

The following equation shows how NCGFS uses deep reinforcement learning to minimise the Loss $L$ for a given environment s to produce an optimal forecast from the probabilistic distribution of forecasts $\pi = P(f|s)$.

$$Q^*(s,\ f) = \min_{\pi} \frac{1}{n}\left(\sum_{i}^{n} \gamma^i L_{t+i} \mid s_t = s,\ f_t = f,\ \pi\right)$$

## Recurrent Optimization Algorithms

RMSProp was found empirically to be the optimal algorithm for optimization of the proposed model. Adam was not selected as the dataset size was small and the algorithm did not converge.

Table S1. Adapted summary of optimization algorithms considered.[2]

| Method | Properties | Advantages | Disadvantages |
|---|---|---|---|
| GD | Solve the optimal value along the direction of the gradient descent. The method converges at a linear rate. | The solution is global optimal when the objective function is convex. | In each parameter update, gradients of total samples need to be calculated, so the calculation cost is high. |
| SGD | The update parameters are calculated using a randomly sampled mini-batch. The method converges at a sublinear rate. | The calculation time for each update does not depend on the total number of training samples, and a lot of calculation cost is saved. | It is difficult to choose an appropriate learning rate, and using the same learning rate for all parameters is not appropriate. The solution may be trapped at the saddle point in some cases. |
| AdaGrad | The learning rate is adaptively adjusted according to the sum of the squares of all historical gradients. | In the early stage of training, the cumulative gradient is smaller, the learning rate is larger, and learning speed is faster. The method is suitable for dealing with sparse gradient problems. The learning rate of each parameter adjusts adaptively | As the training time increases, the accumulated gradient will become larger and larger, making the learning rate tend to zero, resulting in ineffective parameter updates. A manual learning rate is still needed. It is not suitable for |

For peer review only

| | | | |
|---|---|---|---|
| | | | dealing with non-convex problems |
| AdaDelta/ **RMSProp** | Change the way of total gradient accumulation to exponential moving average. | Improve the ineffective learning problem in the late stage of AdaGrad. It is suitable for optimizing non-stationary and non-convex problems. | In the late training stage, the update process may be repeated around the local minimum. |
| Adam | Combine the adaptive methods and the momentum method. Use the first-order moment estimation and the second order moment estimation of the gradient to dynamically adjust the learning rate of each parameter. Add the bias correction. | The gradient descent process is relatively stable. It is suitable for most non-convex optimization problems with large data sets and high dimensional space. | The method may not converge in some cases. |

## Neural Network architecture and configuration

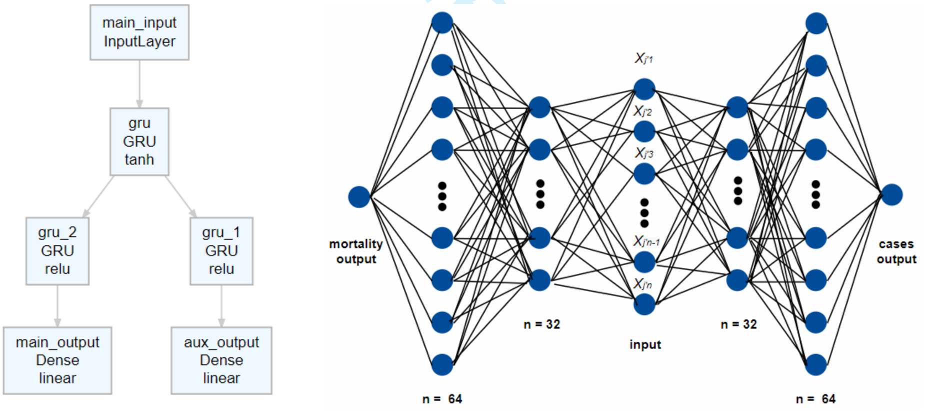


Figure S4.1 NCGFS model architecture. Note, the two layers on either side of the input layer have been depicted in this manner to facilitate representation but represent the same layer.

The model is comprised of symmetrical neuro network that consists of six layers. The input layer accepts a data matrix in the dimension of $[d \quad \tau \quad n]$, where $d$ = 136 is the number of variables, $\tau$ = 2 is the number of time steps in the recurrent direction along the hidden layer, $n$ is the number of samples taken from the observation. The

layer immediately right of the input layer is named gru and consists of 32 GRU cells or units. The next layer to the right is name gru_2 and consists of 64 GRU cells. The output from this layer uses the ReLU activation function as this has been demonstrated to be effective for the convergence of neuro networks. This is followed by a dense output layer named main_output, consisting of one unit on the right. This layer provides the prediction output for the number of COVID-19 cases five weeks ahead of the corresponding weeks in the input data.

The layer immediately left of the input layer is in fact the same layer as that to the right of input layer i.e. named gru. The next layer to the left is a named gru_1 and consists of 64 GRU cells. This is followed by a dense output layer named aux_output for prediction the number of mortalities five weeks ahead of the corresponding weeks in the input data.

We have empirically found that this combination of depth and width is efficient for the minimising the loss of the learning problem at hand.

Model-M was trained using four iterations of 500 epochs (with 6 steps per training epoch and 1 step per validation epoch) using early stop to stop training once validation loss has stopped decreasing with a difference of 0.001 and patience of 200. With the four iterations of training, NCGFS model-M had achieved a performance validation loss of 0.17456 per epoch consisting of the sum of validation loss for both cases and mortality.

Part II. A

1. File 2: domains of deprivation

url location:

https://www.gov.uk/government/statistics/english-indices-of-deprivation-2019

For peer review only

2019 indices containing individual metrics including health, education

Index of Multiple Deprivation (IMD) Decile (where 1 is most deprived 10% of LSOAs)

IMD Rank (1 is most deprived)

2. 2001 to 2018 edition of this dataset

Public houses and bars by local authority

url location:

https://www.ons.gov.uk/businessindustryandtrade/business/activitysizeandlocation/datasets/publichousesandbarsbylocalauthority

Using Pubs size LA 2018 by local authority. Gives the number of pubs in UK by local authority

3. 2020 edition of this dataset

UK business: activity, size and location

url location:

https://www.ons.gov.uk/businessindustryandtrade/business/activitysizeandlocation/datasets/ukbusinessactivitysizeandlocation

ukbusinessworkbook2020.csv by Retail, Transport_Storage_inc_postal, Accommodation_food services, Education, Health, Arts_entertainment recreation_other_services

4. Local Authority Districts 2019 boundaries

Local Authority Districts in the United Kingdom, as at 31 December 2019. The boundaries available are:

- (BUC) Ultra Generalised (500m) - clipped to the coastline (Mean High Water mark).

https://geoportal.statistics.gov.uk/datasets/local-authority-districts-december-2019-boundaries-uk-buc

We use this as COVID-19 cases data is based on 2019 LA boundaries

For peer review only

5. Local Authority District to Public Health England Centre to Public Health England Region (December 2019) Lookup in England

url:

https://geoportal.statistics.gov.uk/datasets/local-authority-district-to-public-health-england-centre-to-public-health-england-region-december-2019-lookup-in-england

6. Mid-2019: April 2019 local authority district codes edition of this dataset

Population estimate data

url:
https://www.ons.gov.uk/peoplepopulationandcommunity/populationandmigration/populationestimates/datasets/populationestimatesforukenglandandwalesscotlandandnorthernireland

Using the following sheets: MYE2 – Males, MYE2 – Females, MYE3 (migrationFlow), MYE2 – Persons (contains age population).

7. SchoolOpening

url:

https://www.gov.uk/government/publications/actions-for-schools-during-the-coronavirus-outbreak/guidance-for-full-opening-schools

https://www.bbc.co.uk/news/uk-51952314

Schools have remained open to some pupils since 23 March, welcoming more pupils back from 1 June.

During model generation, the SchoolOpening index was linked to the main dataset based on the weeks the school restriction policies were implemented and the relative effects at each time period. Index definition: 3 = No restrictions; 1.5 = School closing but remaining open to some pupils; 3 = Also used to represent school reopening.

Unlike the LockdownScore indices, the SchoolOpening index already takes into account of the effect that school closing typically occurs when the number of

For peer review only

cases or mortality is highest, by inversing scoring. Hence, in this case when varying the SchoolOpening parameter during model configuration mode, this metric will not have an inverse effect.

Note, the effects pertain only to the local authorities selected.

8. Number of monthly arrivals in tourist accommodation in Spain from August 2018 to July 2020*

url:

https://www.statista.com/statistics/1130775/number-of-monthly-arrivals-short-stay-accommodation-in-spain/

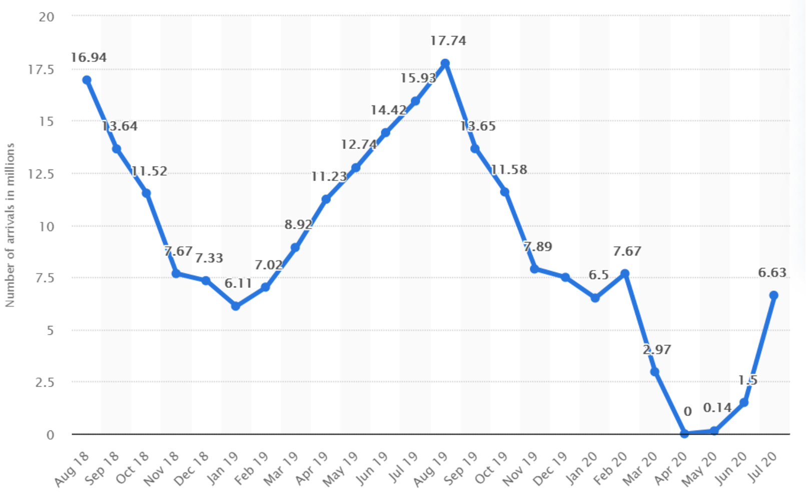


Figure S4.2. Monthly worldwide tourist arrivals in accommodation in Spain

We adjust tourist arrival by the proportion of UK citizens travelling to Spain in 2019 (section 9.)

For peer review only

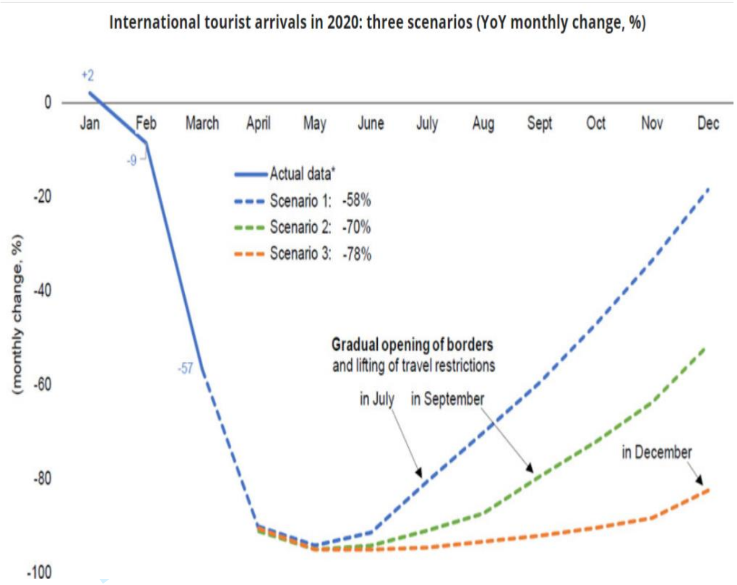


Figure S4.3 A reference model for understanding how quarantine measures are modelled.

url: https://www.adpr.co.uk/blog/covid-19/travel-and-tourism-brands-can-recover-from-coronavirus/

As UK removed 75 countries from quarantine list in July, we impute missing values from August to October based on the recovery model for July opening of borders above. However, the actual recovery based on the data is much faster than that shown in figure above.

9. Number of international tourists arriving in Spain in 2019, by country of residence

url:

https://www.statista.com/statistics/447683/foreign-tourists-visiting-spain-by-country-of-residence/

Obtains proportion of UK citizens arriving in Spain in 2019 as estimate for proportion in 2020

statistic_id447683_international-tourist-arrivals-in-spain-2019-by-country-of-residence.xlsx

UK proportion: 0.215984348

This is the estimated number of tourists arriving in Spain from UK in millions. The values are calculated based on number of tourists from various countries arriving in Spain from Jan 2020 to July 2020 and then adjusting by multiplying this number by the proportion of UK tourists in Spain from the preceding year i.e. 2019.

The weeks not covered by the data available i.e. August to October are imputed based on the tourism industry recovery model for July opening of borders https://www.adpr.co.uk/blog/covid-19/travel-and-tourism-brands-can-recover-from-coronavirus/.

This metric should be used as a guidance for how the situation will vary when international travel is restricted.

10. Public Health England Centres (December 2016) Ultra Generalised Clipped Boundaries in England

url:

https://geoportal.statistics.gov.uk/datasets/public-health-england-centres-december-2016-ultra-generalised-clipped-boundaries-in-england

2016 is the latest available

11. QuarantineMeasures

url:

https://www.theweek.co.uk/107044/UK-coronavirus-timeline+&cd=4&hl=en&ct=clnk&gl=uk

During model generation, the QuarantineMeasures index were linked to the main dataset based on the weeks the travel quarantine policies were implemented and the relative effects at each time period. Index definition: 1 = No quarantine; 10 = full quarantine of tourist from all countries; 5 = Removal of 59 countries from quarantine

For peer review only

list; 5.5 = Adding Spain back to the quarantine list following removal of 59 countries from the list.

As the level of travel quarantine restrictions implemented is dependent on the severity of covid-19 situation in other countries rather than the number of cases and mortality in UK, when varying this parameter during model configuration mode, this metric will have not an inverse effect.

Note, the effects pertain only to the local authorities selected.

12. LockdownScore

During model generation, the LockdownScore index were linked to the main dataset based on the weeks the lockdown policies were implemented and the relative effects at each time period. Index definition: 1 = No lock down; 4 = Local lock down; 5 = Local lock down with social distancing; 10 = Full Lock Down.

However, as the level of lock down implemented is typically highest when the number of cases or mortality is highest, when varying this parameter during model configuration mode, this metric will have an inverse effect. That is, changing the LockdownScore value to 1 will result in a full lock down, whilst changing to 10 will result in no lock down. 4 will result in Local lock down with social distancing and 5 will represent Local lock down.

Note, the effects pertain only to the local authorities selected.

For peer review only

Part II. B

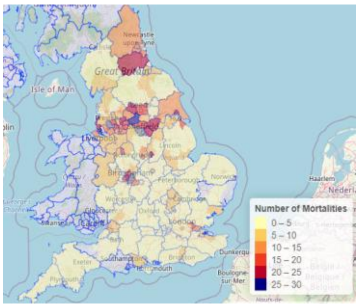


(i) M: Actual Mortalities Wk 46

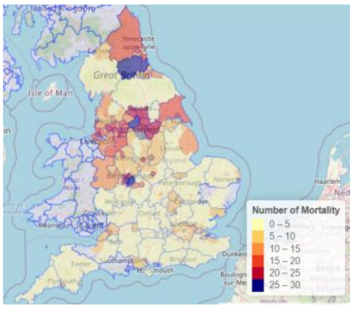


(ii) M: Wk 51 Local LD_SD

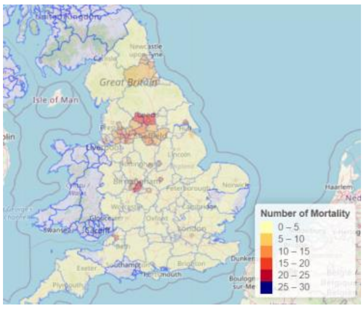


(iii) M: Wk 51 FLD

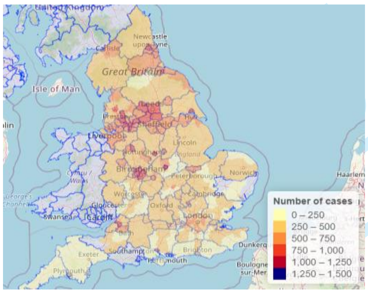


(iv) C: LD_SD -50% school

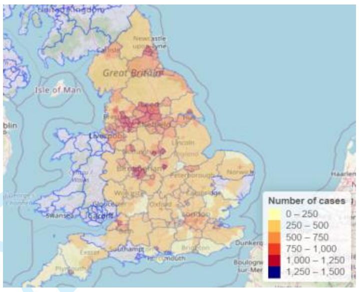


(v) C: LD_SD -50% food & accom

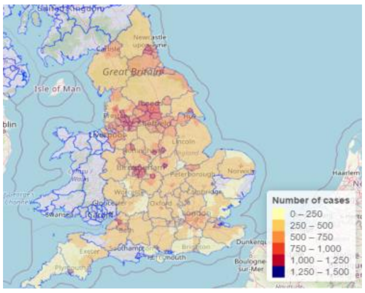


(vi) C: LD_SD -50% retail

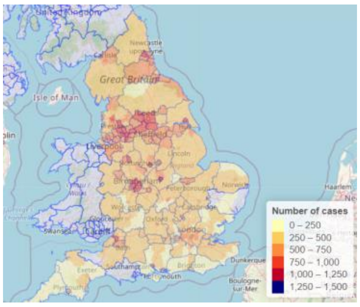


(vii) C: LD_SD -50% pubs

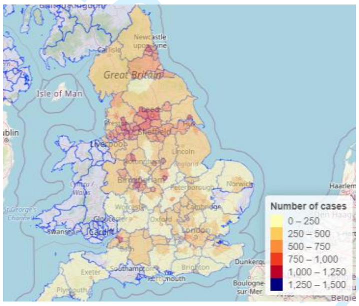


(viii) C: LD_SD intl travel -50%

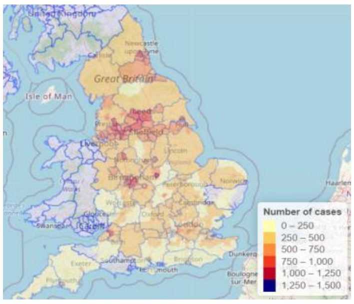


(ix) C: LD_SD 100% quarantine

Figure S5. Geographical level of cases and mortality for actual and predicted results based on different measures. C: cases; M: mortalities.

Part III

Table S2. Internal and external validation metrics for NCGFS compared against models from other studies.

| Models | Predicted variables | RMSE | MAE | MAPE | Correlation Coefficient |
|---|---|---|---|---|---|
| | Internal validation (week 46 FLD) | | | | |
| NCGFS (England) | Cases | 152.24 | 80.47 | 0.3649 | 0.81 |
| Devaraj et al. SLSTM (India) | Cases | 274.22 | 920.02 | 0.3 | 1.00 |
| Melin et al. MNNF (Mexico) | Cases | 1554.03 | - | - | - |
| NCGFS (England) | Mortality | 4.70 | 2.72 | NaN* | 0.76 |
| Devaraj et al. SLSTM (India) | Mortality | 309.12 | 278.29 | 0.6 | 1.00 |
| Melin et al. MNNF (Mexico) | Mortality | 170.00 | - | - | - |
| | External validation (week 51 LD_SD) | | | | |
| NCGFS (21 hotspots) | Cases | 798.83 | 721.42 | 1.23 | 0.27 |
| NCGFS (21 hotspots) | Mortality | 11.94 | 8.84 | 0.53 | 0.46 |
| | External validation (week 51 FLD) | | | | |
| NCGFS (21 hotspots) | Cases | 700.88 | 453.05 | 0.46 | 0.42 |
| NCGFS (21 hotspots) | Mortality | 14.91 | 10.05 | 0.39 | 0.68 |

* it was not possible to calculate MAPE here due to low LA rates and presence of zeros.

Table S3. Ranking of model compared to those from Devaraj et al.[3]

| Model | Average Ranking | Overall Rank |
|---|---|---|
| NCGFS (England internal validation) | 0.570 | 1 |
| NCGFS (21 hotspots external validation FLD) | 0.996 | 2 |
| NCGFS (21 hotspots external validation LD_SD) | 1.206 | 3 |
| Devaraj et al. SLSTM (India) | 1.753 | 4 |
| Devaraj et al. ARIMA (India) | 1.910 | 5 |
| Devaraj et al. LSTM (India) | 2.113 | 6 |

For peer review only

Part IV.

The deep learning model was used to generate the plots for fig S5. of the effects on the predicted number of cases for Southampton based on different measures.

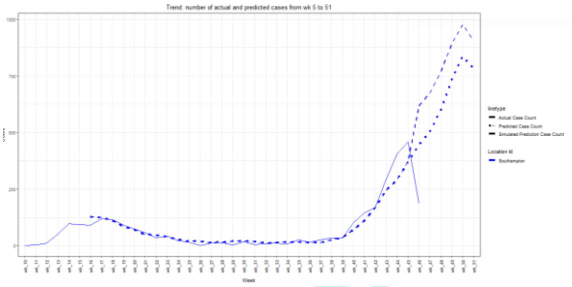


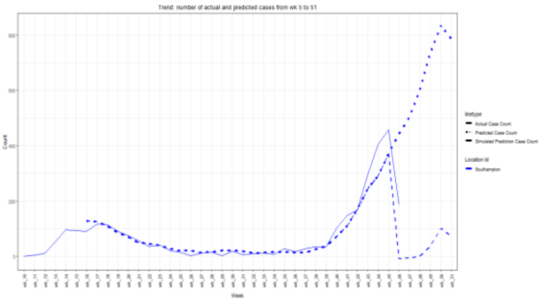


(a) No lockdown vs. LD_SD

(b) LD_SD vs. full lockdown

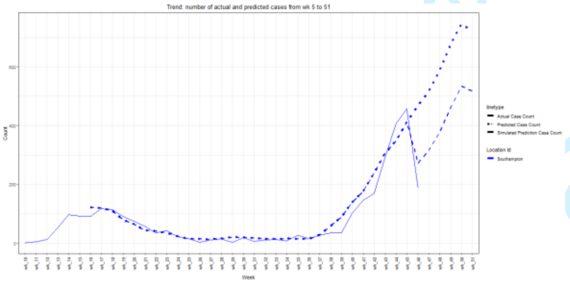


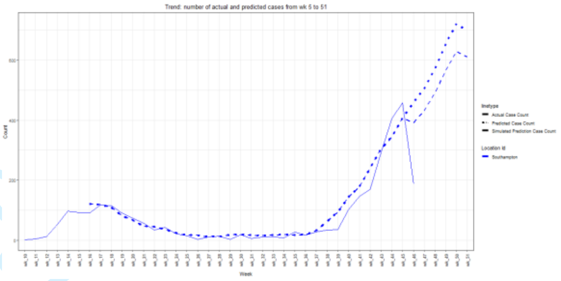


(c) LD_SD vs. international travel -50%

(d) LD_SD vs. closing school -50%

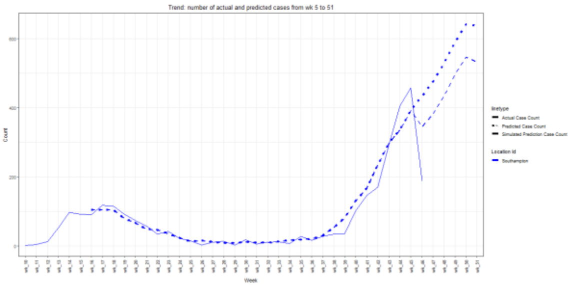


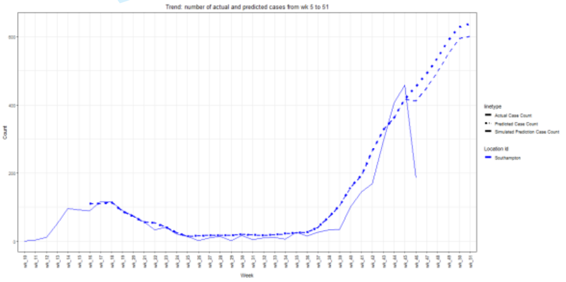


(e) LD_SD (quarantine 5.5) vs. full quarantine (10)

(f) LD_SD (100% pubs) vs. -50% pubs

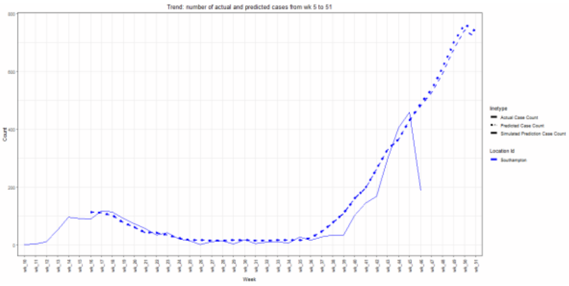


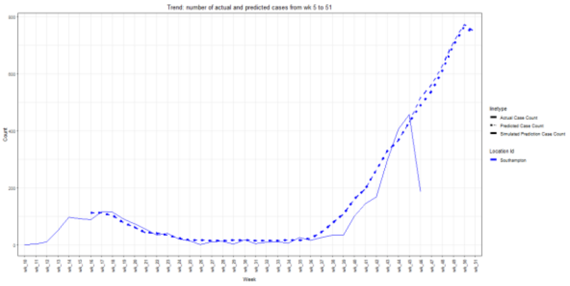

(g) LD_SD (100% food & Accom) vs. -50%

(h) LD_SD (100% Retail) vs. -50% Retail

Figure S6. Cases forecast for Southampton by measure. The dotted line represents predictions using the LD_SD measures. The dashed lines represent prediction changes based on changes to measures. (c-h) relate to LD_SD without (left) and with (right) the supplementary measures.

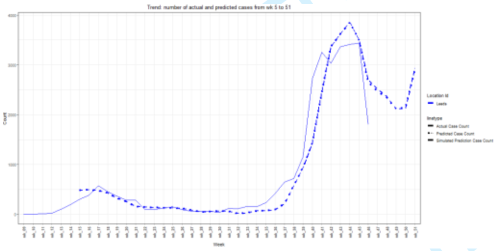

(a) No lockdown vs. LD_SD

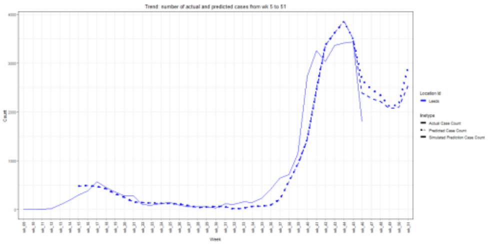

(b) LD_SD vs. full lockdown

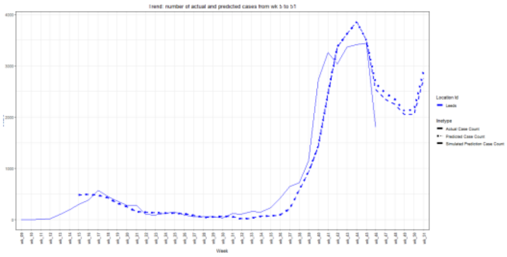

(c) LD_SD vs. international travel -50%

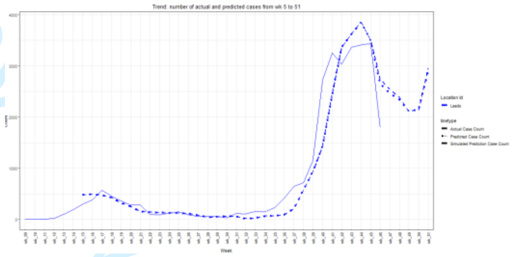

(d) LD_SD vs. closing school -50%

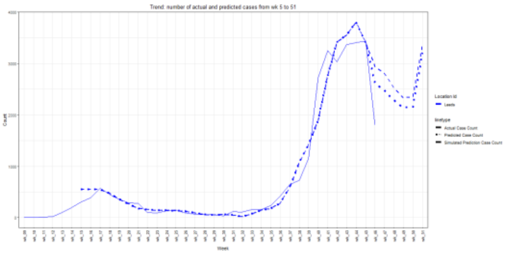

(e) LD_SD (quarantine 5.5) vs. full quarantine (10)

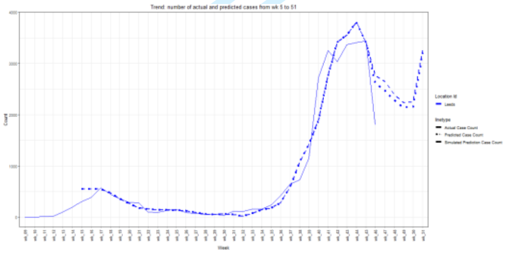

(f) LD_SD (100% pubs) vs. -50% pubs

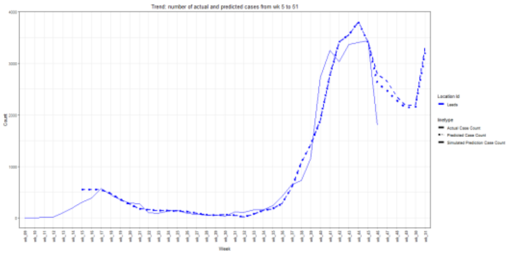


(g) LD_SD (100% food & Accom) vs. -50%

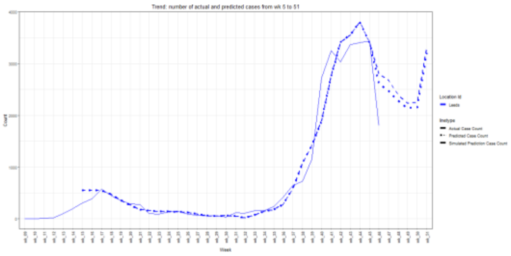


(h) LD_SD (100% Retail) vs. -50% Retail

Figure S7. Cases forecast for Leeds by measures. The dotted line represents predictions using the LD_SD measures. The dashed lines represent prediction changes based on changes to measures. (c-h) relate to LD_SD without (left) and with (right) the supplementary measures.

## References

1 Mnih V, Kavukcuoglu K, Silver D, *et al.* Human-level control through deep reinforcement learning. *Nature* 2015;**518**:529–33. doi:10.1038/nature14236

2 Sun S, Cao Z, Zhu H, *et al.* A Survey of Optimization Methods from a Machine Learning Perspective. *ArXiv190606821 Cs Math Stat* Published Online First: 23 October 2019.http://arxiv.org/abs/1906.06821 (accessed 24 Nov 2021).

3 Devaraj J, Madurai Elavarasan R, Pugazhendhi R, *et al.* Forecasting of COVID-19 cases using deep learning models: Is it reliable and practically significant? *Results Phys* 2021;**21**:103817. doi:10.1016/j.rinp.2021.103817

For peer review only

TRIPOD Checklist: Prediction Model Development

| Section/Topic | Item | Checklist Item | Page |
|---|---|---|---|
| **Title and abstract** | | | |
| Title | 1 | Identify the study as developing and/or validating a multivariable prediction model, the target population, and the outcome to be predicted. | 1 |
| Abstract | 2 | Provide a summary of objectives, study design, setting, participants, sample size, predictors, outcome, statistical analysis, results, and conclusions. | 2 |
| **Introduction** | | | |
| Background and objectives | 3a | Explain the medical context (including whether diagnostic or prognostic) and rationale for developing or validating the multivariable prediction model, including references to existing models. | 3 |
| | 3b | Specify the objectives, including whether the study describes the development or validation of the model or both. | 4 |
| **Methods** | | | |
| Source of data | 4a | Describe the study design or source of data (e.g., randomized trial, cohort, or registry data), separately for the development and validation data sets, if applicable. | 6-7 |
| | 4b | Specify the key study dates, including start of accrual; end of accrual; and, if applicable, end of follow-up. | 8 |
| Participants | 5a | Specify key elements of the study setting (e.g., primary care, secondary care, general population) including number and location of centres. | 4 |
| | 5b | Describe eligibility criteria for participants. | 4 |
| | 5c | Give details of treatments received, if relevant. | 4 |
| Outcome | 6a | Clearly define the outcome that is predicted by the prediction model, including how and when assessed. | 8-9 |
| | 6b | Report any actions to blind assessment of the outcome to be predicted. | NA |
| Predictors | 7a | Clearly define all predictors used in developing or validating the multivariable prediction model, including how and when they were measured. | 8-9 |
| | 7b | Report any actions to blind assessment of predictors for the outcome and other predictors. | NA |
| Sample size | 8 | Explain how the study size was arrived at. | 8-9 |
| Missing data | 9 | Describe how missing data were handled (e.g., complete-case analysis, single imputation, multiple imputation) with details of any imputation method. | Supp. Mat. Part V. |
| Statistical analysis methods | 10a | Describe how predictors were handled in the analyses. | 8-9 |
| | 10b | Specify type of model, all model-building procedures (including any predictor selection), and method for internal validation. | Supp. Mat. Part I. |
| | 10d | Specify all measures used to assess model performance and, if relevant, to compare multiple models. | 4-5, 8 |
| Risk groups | 11 | Provide details on how risk groups were created, if done. | NA |
| **Results** | | | |
| Participants | 13a | Describe the flow of participants through the study, including the number of participants with and without the outcome and, if applicable, a summary of the follow-up time. A diagram may be helpful. | NA |
| | 13b | Describe the characteristics of the participants (basic demographics, clinical features, available predictors), including the number of participants with missing data for predictors and outcome. | NA |
| Model development | 14a | Specify the number of participants and outcome events in each analysis. | NA |
| | 14b | If done, report the unadjusted association between each candidate predictor and outcome. | 9-11 |
| Model specification | 15a | Present the full prediction model to allow predictions for individuals (i.e., all regression coefficients, and model intercept or baseline survival at a given time point). | 10-14 |

For peer review only

| | | | |
|---|---|---|---|
| | 15b | Explain how to the use the prediction model. | Supp. Mat. Part II. |
| Model performance | 16 | Report performance measures (with CIs) for the prediction model. | 11, 14 |
| **Discussion** | | | |
| Limitations | 18 | Discuss any limitations of the study (such as nonrepresentative sample, few events per predictor, missing data). | 10 |
| Interpretation | 19b | Give an overall interpretation of the results, considering objectives, limitations, and results from similar studies, and other relevant evidence. | 15-17 |
| Implications | 20 | Discuss the potential clinical use of the model and implications for future research. | 16-17 |
| **Other information** | | | |
| Supplementary information | 21 | Provide information about the availability of supplementary resources, such as study protocol, Web calculator, and data sets. | Supp. Mat. |
| Funding | 22 | Give the source of funding and the role of the funders for the present study. | 21 |

We recommend using the TRIPOD Checklist in conjunction with the TRIPOD Explanation and Elaboration document.

For peer review only
